# Transmission of human handedness: a reanalysis

**DOI:** 10.1017/ehs.2026.10038

**Published:** 2026-02-16

**Authors:** Rony Karstadt, Chloe Shiff, Tomer Oron, Nadav Ben Nun, Yoav Ram

**Affiliations:** 1 School of Zoology, Faculty of Life Sciences, Tel Aviv University, Tel Aviv, Israel; 2 Institute for Computational and Mathematical Engineering, Stanford University, Stanford, CA, USA; 3 Safra Center for Bioinformatics, Tel Aviv University, Tel Aviv, Israel; 4 Sagol School of Neuroscience, Tel Aviv University, Tel Aviv, Israel

**Keywords:** handedness, cultural evolution, gene-culture co-evolution, human evolution

## Abstract

Human handedness results from the interplay of genetic and cultural influences. A gene-culture co-evolutionary model for handedness was introduced by Laland et al. (1995), and this study generalizes that model and the related analysis. We address ambiguities in the original methodology, particularly regarding maximum-likelihood estimation, and incorporate sex differences in cultural transmission. By fitting this extended framework to existing familial and twin datasets, we demonstrate that accounting for criterion shifts significantly improves model fit and parameter estimation accuracy. We find stronger maternal than paternal effects on handedness, with daughters exhibiting greater sensitivity to these effects than sons. We provide an open-source Python implementation of the model, which is a robust platform for comparing gene-culture models and applying them to diverse datasets.

## Graphical abstract

**Figure:**
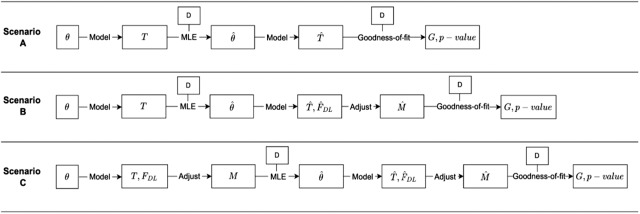

## Introduction

About 90% of humans are right-handed (e.g., Corballis, 1991), and the handedness dimorphism is consistent across the hominin lineage (Uomini & Ruck, 2018), including in modern societies (Raymond & Pontier, 2004). The predominance of right-handedness has sparked considerable interest in the evolutionary dynamics underlying human handedness. Over the years, many studies have addressed the evolutionary mechanisms that might underlie this trait (McManus, 2019).

Human hand usage encompasses two primary traits often indiscriminately referred to as ‘handedness’, but with a crucial distinction between them. *Hand preference* describes the habitual choice of hand usage for one-handed tasks, while *hand performance* identifies the more effective hand for a task, irrespective of the hand that commonly executes the task. Different studies employ differing measurements for *hand preference* and *hand performance* (Janßen, 2004), and this discrepancy has been considered responsible for the variation in observed left-handedness prevalence between studies and generations (Peters, 1998). Thus, it is difficult to build on the results of experiments on handedness to address how human hand usage patterns evolved.

Mendelian genetic models of human handedness have struggled to account for the inheritance pattern observed in family studies. Such studies found that two right- or left-handed parents have about 63% or 11% chance of having right- or left-handed offspring (Porac, 2016). Yet, simple two-allele Mendelian models (Annett, 1964; Chamberlain, 1928; Ramaley, 1913; Rife, 1950; Trankell, 1955) with dominance of the right-handed allele cannot reproduce these proportions: although they allow left-handed offspring from two right-handed heterozygote parents, they still predict stronger parent-offspring similarity than observed. Similarly, such models predict 100% concordance between monozygotic twins, or at least greater concordance than dizygotic twins. However, again, studies found a concordance of around 80% for both dizygotic and monozygotic twins (Pfeifer et al., 2022). Furthermore, research on twins and their non-twin siblings found that a shared family effect has little contribution to predicting handedness (Morgan & Corballis, 1978). These studies thus suggest that genetics constitutes only one factor in determining handedness, but the size of its contribution is still unclear (Schmitz et al., 2017).

From the subtly right-biased bell-curved distribution of hand performance, Annett (1972, 1975, 1978) suggested a genetic model in which hand preference is a continuous trait determined randomly during development. A ‘right-shift’ allele increases the probability of right- vs. left-handedness (rather than deterministically determining right- or left-hand preference). To accommodate twin data, she also assumed that the right-shift allele is expressed differently in twins (Annett, 1994), and given the available data, she concluded that the observed polymorphism is due to heterozygote advantage (Annett, 1995).

McManus (1985) presented the Dextral-Chance (DC) model that assumes a single gene with two alleles: D (for dextrality) that results in 100% right-handers when homozygous and C (for chance) that results in purely random asymmetry when homozygous, that is, 50% right-handers and 50% left-handers. The degree of dominance of D over C and the true incidence of left-handers were inferred from the data using maximum-likelihood estimation. The variation in the *measured incidence* across studies was assumed to arise due to inconsistent methodological approaches, which he accounted for by adjusting the *measured incidence* to the *true incidence*. His results estimated a *true incidence* of left-handers at 7.75% with 25% of heterozygotes being left-handed. This model, too, suggests that the dimorphism in humans is preserved through heterozygote advantage.

Another factor that could influence handedness is culture (Corballis, 1991). Comparative studies have revealed variations in left-handedness prevalence among different cultures, seemingly unrelated to geographic proximity (McManus, 2009). In a meta-analysis, Papadatou-Pastou et al. (2020) used the writing hand as an indicator for *hand preference* and found a 9.29% prevalence of left-handedness; behavioural observations resulted in 15.11% and responses to a questionnaire ranged from 9.75% to 13.51%. Left-handers also vary according to their ancestry: ‘Europeans’ have the highest potential to be left-handed (11.12%), followed by ‘sub-Saharan Africans’ (7.71%) and ‘East Asians’ (5.69%). However, even in the most permissive estimates, left-handedness rates ranged from 0% to 30% only (Marchant & McGrew, 1998), and no evidence has been found for a culture in which the frequency of left-handers outnumbers or equals that of right-handers. Studies published before 1975 indicated a 7.2% prevalence of left-handers, but in the following years this percentage increased up to a peak of 11.7% between 1996 and 2007, followed by a decline to 10.8% until 2019 (Papadatou-Pastou et al., 2020).

Examining various tasks humans perform with their hands has revealed that culture has a nuanced influence on hand preference. It is not uncommon to find that individuals consistently use their left hand but write with the right, a phenomenon more prevalent in early-birth cohorts and specific cultures (Medland et al., 2004). A central hypothesis for this discrepancy is that a change in cultural norms has led to the acceptance of left-handed writing in schooling (Harris, 1980). This cultural change can account for the increase in the prevalence of left-handed writing over the past century in many western countries (e.g., Beukelaar & Kroonenberg, 1986; Brackenridge, 1981). In countries in which left-handed writing is still discouraged, an increase in the prevalence of left-handers has not been observed (Shimizu & Endo, 1989). Porac and Coren (1981) pointed out that predominantly right-handed societies tend to motivate left-handers to conduct tasks with their right hand due to physical constraints (e.g., scissors, computer mouse, knife) or conformity (e.g., writing, cutlery), even if this goes against their natural inclination. Furthermore, evidence from non-western societies with a low literacy rate does not indicate a right-hand bias for any hand-based task other than a weak but consistent tendency in tool use (Marchant et al., 1995). Therefore, it may be that modern humans are somewhat committed to use their right hand in certain tasks, with the level of commitment varying across tasks and cultures.

Genomic-era evidence shows modest but consistent genetic contributions to handedness. Twin meta-analyses estimate additive genetic effects of ∼25% with negligible shared family environment effect (Medland et al., 2006; Paracchini, 2021). SNP-based heritability for left-handedness is low, ∼4.35% in UK Biobank (De Kovel et al., 2019). Large-scale genome-wide association studies demonstrate highly polygenic architecture with many small-effect loci (Cuellar-Partida et al., 2020). Rare coding variants contribute an exome-wide heritability of ∼0.9% (Schijven et al., 2024). These findings suggest that genetic effects exist but are weak, polygenic, and insufficient to account for cross-cultural and familial variation alone.

The only model that sought to account for gene-culture evolution of human handedness was published by Laland et al. (1995). Following the genetic model by McManus (1985), they assumed that the tendency of an individual towards one of the two phenotypes – right- and left-handedness – depends on a single gene with alleles D for a predisposition towards right-handedness and C for lack of such predisposition. They also included a cultural vertical effect of parent phenotype on offspring phenotype, so the probability of becoming right-handed depends on the individual genotype (DD, DC, or CC) and the parental phenotypes (R×R, R×L, or L×L). Analysis of the equilibrium allele and phenotype frequencies showed that if natural selection acts on the phenotype or directly on the genotype, allele D will go to fixation unless there is heterozygote advantage; that is, gene-culture interaction will not preserve genetic variation in handedness, and all stable variation in the equilibrium of their model arises from cultural transmission (with the fixed allele only biasing phenotype probabilities.) Laland et al. (1995) used their equilibrium phenotype frequencies to test this model against data. They estimated the model parameters using maximum-likelihood estimation and the adjustment from *measured incidence* to the *true incidence* applied by McManus (1985). Then, they tested its goodness-of-fit using a *G*-test. The analysis was performed on 17 familial datasets previously summarized by McManus (1985). The best-fit model predicted a species-wide 78% genetic predisposition, which increased to 92% in the offspring of two right-handed parents and decreased to 64% in the offspring of two left-handed parents. The model predicted that right-handers should make up 88% of the population (similar to observed frequencies, e.g., Corballis, 1991) and provided a better fit to 16 of 17 familial datasets and 27 of 28 twin datasets compared to previous genetic models (Annett, 1985; McManus, 1985). The model also predicts that the concordance rate in siblings, twins, and unrelated individuals should be ∼80%, which agrees with observations that purely genetic models cannot explain.

Based on these results, Laland et al. (1995) suggested that a history of selective sweeps created a species-wide genetic predisposition for right-hand preference in a facultative rather than obligate manner. Thus, cultural factors operating via parental influence during early childhood development can explain variation within families and across societies. Consequently, human handedness had been ratcheting up towards the proliferation of right-hand bias throughout human evolution due to caregivers socially transmitting their hand preference to children.

Over the past century a substantial literature on handedness has accumulated. Both genes and culture contribute, although their relative contributions remain unresolved, and recent studies call for multifactorial models that integrate both influences (Laland, 2008; Llaurens et al., 2009; Michel et al., 2018; Schmitz et al., 2017). Two prominent post-1995 evolutionary explanations are the *fighting hypothesis*, which maintains left-handedness by negative frequency-dependent selection (Faurie & Raymond, 2005; Raymond et al., 1996), and the *innate-superiority hypothesis* from the sports-performance literature, which proposes frequency-independent perceptual or neural advantages for left-handers in some sports; its evolutionary relevance remains uncertain (Akpinar & Bicer, 2014; Simon et al., 2025). At present, to our knowledge, Laland et al.’s gene-culture model remains the only framework that combines genetic and cultural effects and estimates them from data.

Here, we reproduce Laland et al.’s analysis to establish a foundation for its application and extension. We provide additional detail to aspects of the analysis that were not explicitly discussed in the original paper. We then extend the model to account for differences in parental and offspring sex and compare this extended model to the original model. We provide an open-source implementation written in Python (Van Rossum, 2007) and a transparent replication protocol. This work may provide a foundation for further exploration of gene-culture models and analysis of cross-cultural datasets to test hypotheses that gene-cultural transmission of human handedness, or other traits under gene-culture co-evolution, varies between populations.

## Methods

### Study design

First, we reproduce the analysis of Laland et al. (1995). They studied a gene-culture model for human handedness using maximum-likelihood estimation to estimate model parameters from familial data, testing the model’s goodness-of-fit on the same data using a *G*-test and then testing the model on a separate twin dataset.

The results reported by Laland et al. (1995) are summarized in their Tables 1–3, but some results (log-likelihood values, *G*-test with three model parameters) were not reported. Similar to McManus (1985), they assumed a *criterion shift* and applied an adjustment to model predictions. The details of whether this adjustment was applied during estimation, testing, or both, remain unspecified in the original study.

**Figure 1.**
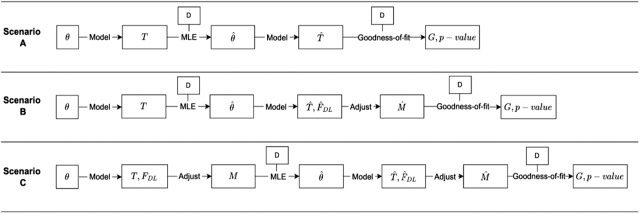
**Study design.** *θ*: model parameters, either *ρ, α* and *β* or *ρ* and *α* when fixing *β* to zero. Model: transformation from parameters to T (Table 1) and from *θ* to *F_DL_* using eqs. 1–2. MLE: parameter inference from data *D* by maximizing *S_T_* (eq. 5; scenario A and B) or *S_M_* (eq. 4; scenario C) using the Nelder–Mead method. Adjust: transform *T* to *M* to adjust for criterion shift using *F_DL_* and eq. 3 (*M = PTO*). Goodness-of-fit: comparing observations and model predictions, that is, *T̂* in scenario A and *M̂* in scenario B and C, using a *G*-test, which results in a *G*-statistic and a *p*-value.

We therefore explored three analysis scenarios (Fig. 1). In scenario A, we estimated model parameters and performed a goodness-of-fit test without applying any adjustment. In scenario B, we estimated the parameters without adjustment but performed a goodness-of-fit test with adjustment. In scenario C, we performed both the estimation and the test with adjustment. Like Laland et al., we examined both a three-parameter and a two-parameter model (see below) in all scenarios to determine if a two-parameter model can account for the observed data as well as a three-parameter model and to produce a protocol for cases in which all three parameters are included in the analysis.

All analyses were implemented in the Python programming language (Van Rossum and others, 2007) with NumPy (Van der Walt et al., 2011), Matplotlib (Hunter, 2007), SciPy (Virtanen et al., 2020), and Pandas (McKinney, 2010). The source code is available at https://github.com/yoavram-lab/Laland1995.

### Familial data

The data used in this study (Table S3) are the same as in Table 3 of Laland et al. (1995). None of the datasets were excluded, and the data were not transformed. The data combine 17 earlier studies published between 1913 and 1985. All 17 studies present data on hand preference (rather than hand performance). 16 of the 17 studies sampled US and UK populations between 1911 and 1980. The frequency of left-handers varies between 3.56% and 24.57%. See supplementary text S1 for more details.

Upon examining the original studies, it became evident that the data as presented in Laland et al. were copied verbatim from McManus (1985): (i) all the data appears in McManus (1985); (ii) we found the same typo in the reference to Chaurasia & Goswami (unpublished); and (iii) the data of Ramaley (1913) is reported incorrectly in both papers, an error likely made by McManus (1985).

Even though the data are presented equivalently in McManus (1985) and Laland et al. (1995), calculating the observed frequency of left-handers in the parent generation, 
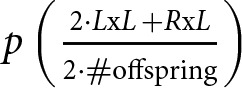
, using the data in Laland et al. did not provide the same frequency as reported by McManus (1985). Indeed, McManus (1985, p. 10) noted that ‘*most of the above studies ignore family size, all the children from a particular family being combined. Thus, if a single RxR pair produced one right- and one left-handed child these two individuals are entered once into column R and once into column L.*’ Thus, the data in McManus (1985) encompass an unknown number of siblings. When we compared the data presented in the original studies to those presented by McManus, we found that the observed frequency had been calculated by McManus using the original data. Hence, alongside the actual data, Laland et al. likely used the observed frequency of left-handers reported by McManus. We validated this assumption by using the observed frequency in the parent generation reported in McManus (1985) and the frequency calculated assuming triplets in the data. Both produced results similar to Laland et al., but the former produced a better match.

### Twin data

We use the 13 twin datasets (Table S10) from Table 4 in Laland et al. (1995), which were taken from McManus (1985) plus an additional dataset from Neale (1988). The data includes the observed numbers of right-right, right-left, and left-left pairs of monozygous (MZ) and dizygous (DZ) twins without parental phenotype. The data totals 2,900 pairs of MZ with a left-handedness rate of 13.8% and a discordance rate of 21.68% and 2,589 pairs of DZ twins with a left-handedness rate of 13.34% and a discordance rate of 22.6%.

### Gene-culture model

Laland et al. (1995) present a gene-culture model for hand preference, categorizing individuals as right- or left-handed without an ambidextrous phenotype. Based on McManus’s (1985) genetic model, alleles D and C at a single locus influence handedness: D increases right-handedness probability, while C lacks this effect. Right-hand probabilities are 
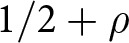
 for DD homozygotes, 
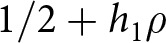
 for DC heterozygotes, and 1/2 for CC homozygotes. Cultural transmission modifies the offspring right-hand probabilities, increasing or decreasing them by *α* when both parents are right- or left-handed, respectively, and by *β* when parents differ (Table S1).

They assumed the ancestral population was CC, with modern handedness shaped by direct or indirect selection on D. Direct selection favours right-handers; indirect selection links D to another lateralized trait. No sex differences in inheritance were considered. Mathematical analysis revealed a single evolutionary trajectory: allele D becomes fixed, eliminating genetic variation in handedness, and phenotypic variation persists due to incomplete genetic effects, 
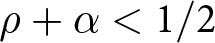
.

The equilibrium frequency of right-handers with allele D (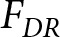) is given by (eq. 3 in Laland et al. 1995)

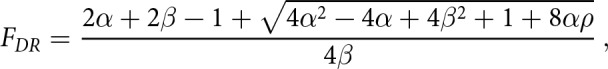
and the frequency of left-handers with the D allele (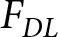) is
(1)
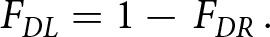


If 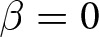, the corresponding equilibria are given by (eq. 3a in Laland et al. 1995)
(2)
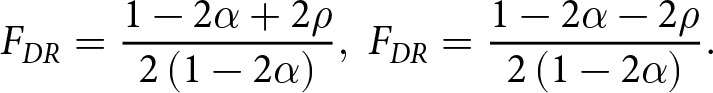


The model, therefore, predicts the *true incidence* of offspring handedness given parental matings in Table 1.

**Table 1.** Frequency of right- and left-handed offspring given parental phenotypes and assuming the *D* allele is fixed in the population, so that the frequency of right-handers in the next *F’_DR_*, given the frequency in the current generation *F_DR_*, follows the recursion *F’_DR_* = *F*^2^*_DR_*(1/2 + *ρ* + *α*) + 2*F_DR_*(1 − *F_DR_*)(1/2 + *ρ* + *β*) +(1 − *F*_DR_)(1/2 + *ρ* − *α*). *Model* row shows the expectations of the model, i.e., the values of matrix *T. Data* row shows a summary of the data. Other rows show the maximum-likelihood estimated model parameters and the corresponding predictions without (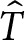) and with adjustment (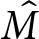) for criterion shift and for both the model with three parameters and the model with two parameters and *β* fixed at zero. See supplementary tables for more details

Parental mating		Right × Right		Right × Left		Left × Left	
Offspring phenotype		Right	Left	Right	Left	Right	Left
Model		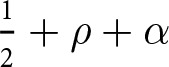	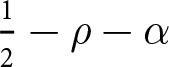	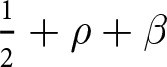	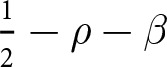	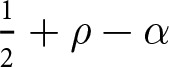	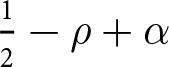
Data		0.915	0.085	0.779	0.221	0.619	0.381
Scenario A & B MLE without adjustment	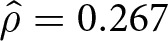 , 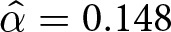 , 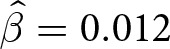	0.915	0.085	0.779	0.221	0.619	0.381
Scenario A & B MLE without adjustment	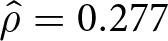 , 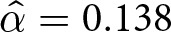 , 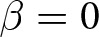	0.915	0.085	0.777	0.223	0.639	0.361
Scenario C MLE with adjustment	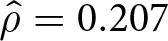 , 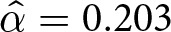 , 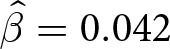	0.91	0.09	0.749	0.251	0.504	0.496
Scenario C MLE with adjustment	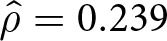 , 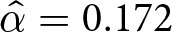 , 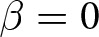	0.911	0.089	0.739	0.261	0.567	0.433
Laland et al. (1995)	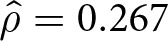 , 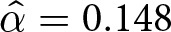 , 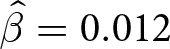	0.915	0.085	0.779	0.221	0.619	0.381
Laland et al. (1995)	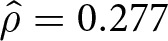 , 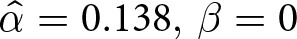	0.915	0.085	0.777	0.223	0.639	0.361

### Adjustment for criterion shift

A long-standing challenge in the field of lateralized hand usage is determining the exact behaviour that defines this trait and how it should be measured (Marchant & McGrew, 1998). This ambiguity results in inconsistent use of methodologies across studies, which, in turn, leads to uncertainty as to whether any observed frequency reflects the population or the particular definition and assessment methodology of left-handedness (Porac, 2016). That is, by using different criteria, one can measure different frequencies of left-handers, an effect termed ‘criterion shift’ (Annett, 1978; McManus, 1985).

### Adjustment in familial data

McManus (1985) proposed the following procedure to address this criterion shift, which was adopted by Laland et al. (1995, Appendix 3). We adjust a matrix *T* of expected frequencies of true right- and left-handed offspring born to true right-handed, left-handed, and mixed-handed parents,

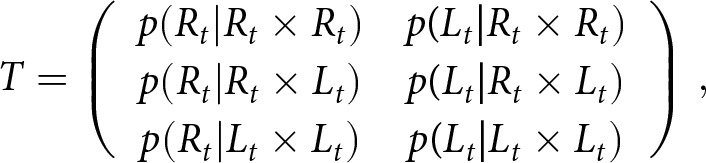
which is determined by the model parameters via Table 2, to a matrix of *M* of expected frequencies of offspring measured as right- or left-handed born to parents measured as right-handed, left-handed, and mixed-handed,

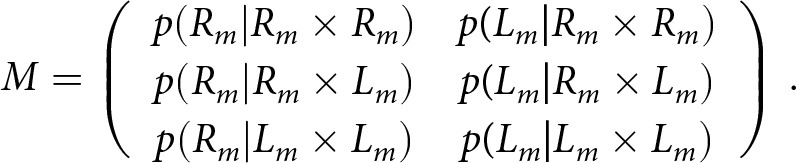

**Table 2.** Comparison of maximum-likelihood estimates to Laland et al. (1995). See supplementary tables for more details

	Maximum-likelihood estimates						
	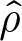	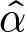	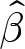	Max. log-lik.	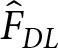	*G*_overall_	# studies with good fit
Laland et al. (1995)	0.267	0.148	0.012	–	–	–	–
	0.277	0.138	0**	–	11.74%	44.43	16
This study							
Scenario A	0.267	0.148	0.012	−8826.643	11.72%	556.7*	9
	0.277	0.138	0**	−8826.793	11.74%	556.0*	9
Scenario B	0.267	0.148	0.012	−8826.643	11.72%	43.6	16
	0.277	0.138	0**	−8826.793	11.74%	44.4	16
Scenario C		0.203	0.042	−8566.939	13.50%	36.3	16
	0.239	0.172	0**	−8567.529	13.56%	37.5	16

* A significant difference between model and data at *p* = 0.05.

** Value fixed at zero.

This procedure assumes a *true incidence* of left handers in the population, *t*, which does not vary across studies and generations. In Laland et al., the model parameters determine this *true incidence* via the equilibrium equations (eqs. 1–2). In any given study, the *measured incidence* of left-handers observed in the data in the parent generation, *m_p_*, and in the offspring generation, *m_o_*, might deviate from the true incidence, *t*, due to criterion shift. Therefore, some true left-handers might be classified as right-handers and vice-versa. These errors are assumed to be mutually exclusive and collectively exhaustive: only one can occur at a time, and it will account for the discrepancy completely.

The parent and offspring generations are adjusted separately, as a discrepancy between *true* and *measured incidence* can occur independently in each generation. *P* and *O* are transition matrices that operate on parent and offspring frequencies, respectively, such that the adjustment is given by
(3)
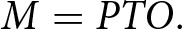


Assuming that the true frequency of each parental mating given the measured one is equal and independent between the parents, the matrix *P* is given by

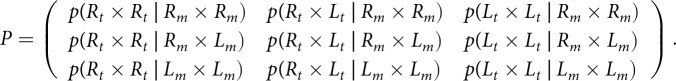


If the *measured incidence* is greater than the *true incidence* in the parent generation, *m_p_ > t*, some true right-handers were classified as left-handers, and no true left-handers were classified as right-handers. Therefore, the proportion of individuals measured as left-handers that are truly right-handers, *u*, is estimated by 
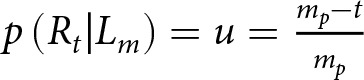
, and the matrix 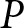 is given by

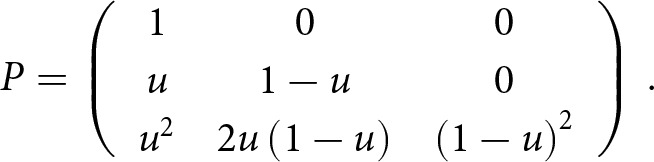


If the *measured incidence* is less than the *true incidence* in the parent generation, *m_p_ < t*, some true left-handers were classified as right-handers, but no true right-handers were classified as left-handers. The proportion of individuals measured as right-handed who are truly left-handed, *v,* is estimated by

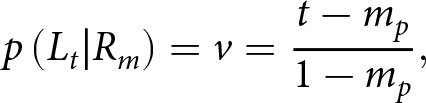
and the matrix P is given by

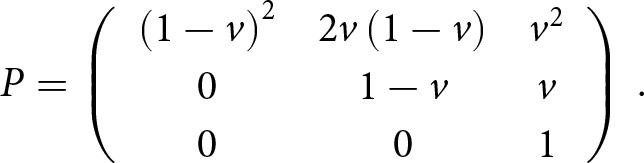


Note that *v* is not explicitly specified in terms of *t* and *m_p_* in Laland et al. (1995); therefore, we deduced it from the matrix *P*.

The matrix *O* gives the probability of an offspring measured as right- or left-handed given that it is truly right- or left-handed and is given by

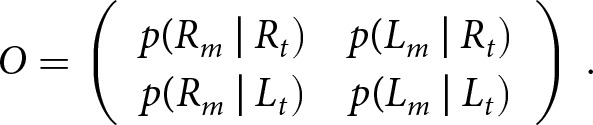


If the *measured incidence* is greater than the *true incidence* in offspring generation, *m_o_ > t*, then the proportion of true right-handers measured as left-handers, *w*, is estimated by

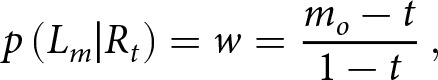
and the matrix 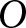 is given by

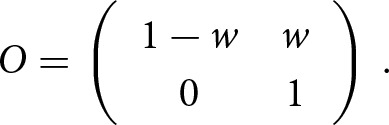


If the *measured incidence* is less than the *true incidence* in offspring generation, *m_o_ < t,* then the proportion of true left-handers measured as right-handers, *x*, is estimated by

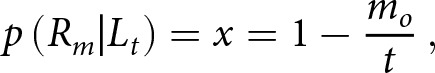
and the matrix 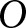 is given by

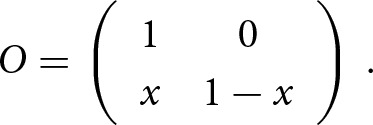


Note that for each dataset, *m_p_* and *m_o_* are computed from the data. Given specific parameter values, *t* is computed from the parameters (eqs. 1–2), compared to *m_p_* and *m_o_*, and then *u* or *v* (but not both) and *w* or *x* (but not both) are computed from *t, m_p_*, and *m_o_*. Thus, *u, v, w,* and *x* are nuisance parameters directly estimated from the model parameters and data summary without maximum-likelihood estimation.

### Adjustment in twin data

The adjustment for criterion shift for twin data is not explicitly described in Laland et al. (1995) but is straightforward. In the twin data the parental phenotype is not explicitly reported, so a single transition matrix, O, is enough. The above matrix describes the probability of measuring the offspring phenotype H_m_, given that the true phenotype is H_t_. For twin data, this matrix is adapted to twins. It describes the probability of measuring the phenotype of twins, H_m_
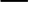
H_m_, given the true phenotype, H_t_
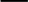
H_t_, where H can be either R for right-handedness or L for left-handedness. Therefore,

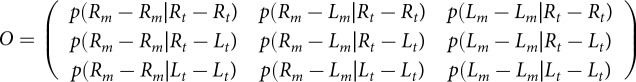


Using the nuisance parameters *w* and *x* described in the adjustment of familial data, in the case of *m_o_ > t,* the matrix *O* is given by

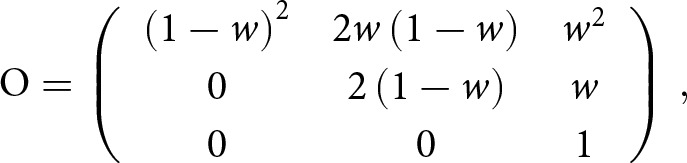
and in the case where *m_o_ < t*, the matrix *O* is given by

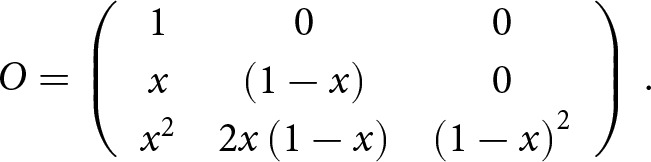


The matrix *T* of expected frequencies of true phenotypes *H_t_*
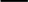
*H_t_* being born to true right-handed, left-handed, and mixed-handed parents,

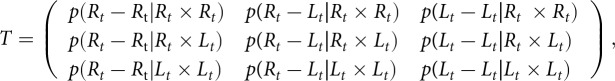
which is determined by the model parameters in Table S2. The model prediction gives the parental phenotype distribution, 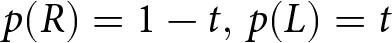. Assuming random mating, we have 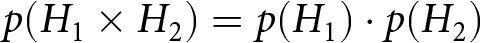. Using the law of total probability, we get the model prediction for the expected frequency of twin phenotypes,

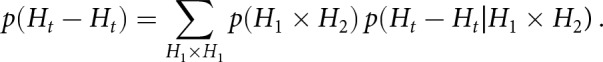


Thus, the *measured incidence* matrix M is given by

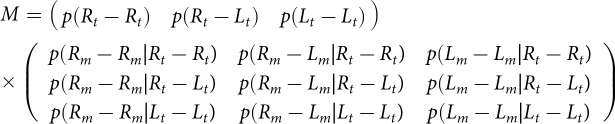


### Likelihood function

The matrices *T* and *M* give the expected frequencies of right- and left-handed offspring born to two right-handed, mixed-handed, and two left-handed parents, without and with adjustment for criterion shift, respectively. Laland et al. (1995) did not explicitly report if they used *T* or *M* for the log-likelihood function (but implied the use of *M* in the definition of S, which they call ‘support function,’ Appendix 3). We therefore examined two log-likelihood functions, *S_T_* and *S_M_*, based on *T* and *M*, respectively.

The log-likelihood function for the model parameters given an observed dataset is the product of the binomial probabilities of right- or left-handed children born to the three parental mating classes. The observed number of right-handed offspring born to two right-handed parents, mix-handed parents, or two left-handed parents is 
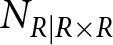
, 
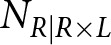
, 
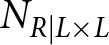
, respectively, with a similar notation for left-handed offspring. Without adjusting for criterion shift, the log-likelihood function is
(4)
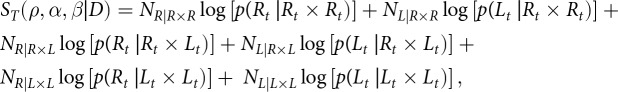
where 
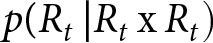
 etc. are the elements of *T*, computed from the model parameters *ρ, α* and *β* using eqs. 1–2. When adjusting for criterion shift, the log-likelihood function is
(5)
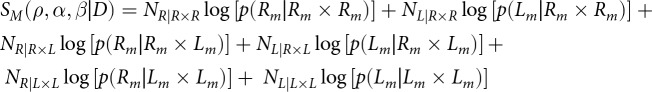
where 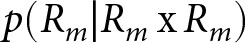 etc. are the elements of *M*, computed from *T, P* and *O* using eq. 3; in turn these are computed from the model parameters *ρ, α* and *β* using eqs. 1–2.

### Statistical inference

Like Laland et al., We inferred the model parameters with all three parameters (*ρ, α,* and *β*) and with only two parameters (*ρ* and *α* while fixing *β* to zero). We then tested for goodness-of-fit using a *G*-test. We performed this analysis for each of the two log-likelihood functions (eqs. 3–4; Fig. 1).

#### Maximum-likelihood estimation

First, we used the Nelder–Mead downhill simplex algorithm implemented in SciPy (*scipy.optimize.minimize*) to minimize the negative log-likelihood function (eqs. 3 or 4). This algorithm was chosen because it does not require gradients of the target function. However, an initial guess is required. To ensure that the result is robust to the initial guess, we used 1,000 random guesses and selected the best result; however, different initial guesses gave similar results. Second, we used a grid search with 1,000 values for each parameter (overall 1,000^3^ parameter combinations) to validate the results and visualize the log-likelihood surface. This procedure was performed to estimate *ρ, α*, and *β*, and again to estimate *ρ* and *α* while fixing *β* to zero. The grid search yielded the same results as the Nelder–Mead algorithm up to three decimal places.

#### Goodness-of-fit test

To test if the model predictions provide a good fit for the observed data, a goodness-of-fit test was performed by computing a *G*-statistic for each study in the dataset (*D_i_*) and across all studies combined (*D*). To determine the contribution of the parameter *β*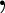 we tested the goodness-of-fit of a model with ρ, α, and β, as and of a model with *β* fixed at zero. Without adjusting for criterion shift, the *G*-statistic is computed from the data *D* and model predictions 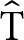 (Table 2 using the MLE parameters) by

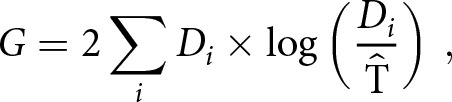
after verifying the following assumptions

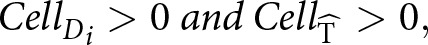

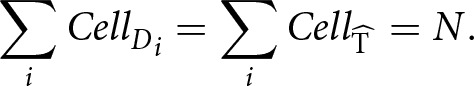

When adjusting for criterion shift, the matrix 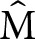 is used instead of the matrix 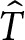 by adjusting 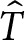 to 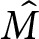 (eq. 3).

***G*-test.** We evaluate the *G*-statistic against a χ^2^ distribution using the *scipy.stats.chi2* function (see supplementary text S2 for details on the degrees of freedom.) A *p*-value less than 0.05 implies a statistically significant result, indicating that the model prediction significantly differs from the observed data and, therefore, the model does not provide a good fit to the data. Note that this test attempts to reject the null hypothesis that the model is correct, and therefore, this test is less useful as support for the correctness and validity of the model. Upon examining the *G*-test results in Laland et al., we observed that the *G*-value across all studies combined was 44.43, not 44.33 as reported. Consequently, we evaluated our results considering the value of 44.43.

### Familial data with sex differences

First, McKeever (2000) compiled data from four different studies from the 1970s and 1980s of primarily college students from the UK and US. This dataset (table 1 in McKeever et al. 2000), which we refer to as the ‘McKeever dataset’, contains data on sex differences in handedness in eight categories: female or male offspring born to two right-handed parents, female or male offspring born to a right-handed mother and left-handed father, female or male offspring born to a left-handed mother and right-handed father, and female or male offspring born to two left-handed parents. In addition, we analyse more recent data collected by Nurhayu et al. (2020) from non-industrialized, agriculture-based societies in the islands of Flores and Adonara, Indonesia. To align it with Laland et al.’s framework, we grouped individuals by generation: the first generation included individuals without children and their siblings; the second generation comprised their parents and their siblings; the third generation contained the grandparents of the first generation and their siblings; and so on, yielding five generations overall and four derived datasets. Because the older generations contained too few left-handed individuals to be informative, we restricted analyses to the first two generations. We call these datasets ‘Generation 1’ and ‘Generation 2’, where the former is the younger generation. Importantly, these datasets contain the sex of all individuals. These data were then represented in triplet form (Table S15). Importantly, the reproduction of Laland et al. (1995) results was conducted solely on the original datasets; the Flores–Adonara dataset was analysed separately to evaluate the model in a different cultural setting and to test the effect of data representation on parameter estimation.

### Extended model with sex differences

We extend the model from Laland et al. (1995) to include sex differences. First, we consider the offspring of a right-handed mother and left-handed father separately from those of a left-handed mother and right-handed father. Thus, we add a model parameter *γ* to represent the cultural effect of a left-handed mother and right-handed father on the probability of a right-handed offspring, while *β* is changed to represent the cultural effect of a right-handed mother and left-handed father on the probability of a right-handed offspring. Second, we consider male and female offspring separately. Hence, the cultural effect parameters differ for female and male offspring, resulting in six parameters: *γ_F_, γ_M,_ β_F_, β_M,_ α_F_,* and *α_M_*. The genetic influence parameter, *ρ,* is the same for female and male offspring. Note that if *α_F_* = *α_M_* and *γ_F_* = *γ_M_ = β_F_* = *β_M_*, then there are no sex differences in handedness, resulting in the three-parameter model of Laland et al. (1995).

- This model extension is described by the probabilities for right-handed female and male offspring given three combinations of alleles and four parental mating types (see Supplementary text S8 and Table S11). We consider different versions of this extended model, depending on whether we assume sex differences between parents and offspring. Together with Laland et al.’s two- and three-parameter models, we have five models: No sex differences, no effect of mixed mating; Laland et al.’s two-parameter model

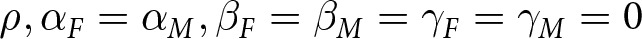

- No sex differences: Laland et al.’s three-parameter model

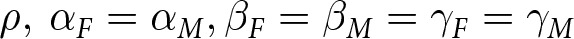

- Sex differences in parents but not in offspring

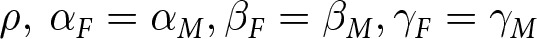

- Sex differences in offspring but not in parents

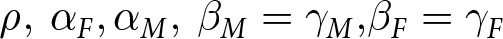

1. Sex differences in both parents and offspring; no constraints on model parameters

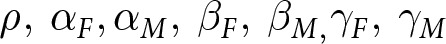


The extended log-likelihood function is
(6)
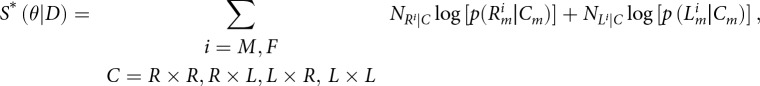
where 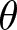 is a vector of model parameters, *i = F* or *M* for female or male offspring, respectively, *R^i^* and *L^i^* are the observed number of right and left-handed offspring of sex *i*, respectively, *C* is an index over four mating types, *R^i^_m_* and *L^i^_m_* are the model predictions for the number of measured right and left-handed offspring of sex *i*, respectively, and *C_m_* is the measured number of parental mating of type *C*.

We estimate parameters for these models using Scenario C, where we adjust for criterion shift before estimating parameters with maximum-likelihood estimation.

### Likelihood-ratio test

We compare the above models I–V using a likelihood-ratio test. Model I is nested in model II; model II is nested in models III and IV; and models III and IV are nested in model V. We compute the likelihood ratio test statistic,

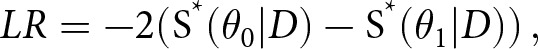
where 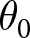 and 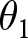 are the parameter vectors of the nested (simple) and nesting (complex) models, respectively. By Wilks’ theorem, *LR* approaches a χ^2^ distribution with *m-n* degrees of freedom, where *n* is the number of parameters in 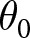 and *m* is the number of parameters in 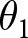 (*m > n*). The conditions for Wilks’ theorem are satisfied because we assume the data is binomially distributed with conditional probability 
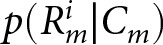
 (Casella & Berger 2002). Therefore, we can calculate a *p*-value for the null hypothesis that the data were generated from the simple nested model parameterized by 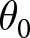, rejecting the null hypothesis in favour of the alternative nesting model parameterized by 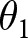 if the *p*-value is sufficiently low.

## Results

### Scenario A: Estimation and testing without adjustment

In this scenario, we estimate the model parameters and test for goodness-of-fit without adjusting for criterion shift for a model with *ρ, α*, and *β* and for a model with *ρ* and *α*, fixing *β* to zero.

**Figure 2.**
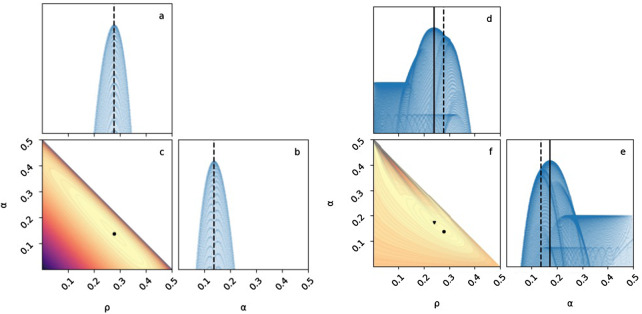
**Maximum-likelihood estimation of two model parameters**. Results of maximum-likelihood estimation (MLE) of *ρ* and *α* when *β* is fixed to zero (see Figures S2 and S3 for the full model). MLE without adjustment (Scenario A and B, circles and dashed lines): 
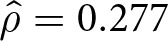
and 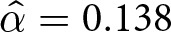 with log-likelihood of −8826.793. MLE with adjustment (Scenario C, triangle and solid lines): 
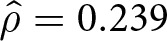
 and 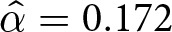 with log-likelihood of −8567.529. Diagonal panels (**a, b, d, e**): Markers show log-likelihood values across 1,000 parameter values (1,000^2^ combinations of *ρ* and *α*). Corner panels (c, f): Contour plots for the joint log-likelihood surface. Point estimates are estimated using the Nelder–Mead algorithm. Contour plots are computed over a grid of 1,000 values for each parameter (same as diagonal panels).

The maximum-likelihood estimates (MLE) of the model parameters are 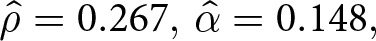 and 
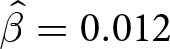
 with a log-likelihood value of −8826.643 (Fig. 2) and a predicted true incidence of left-handedness in the population 
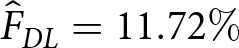
 (eq. 1). When fixing *β* to zero (as the estimate for *β* is small), we estimated values of 
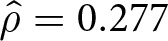
 and 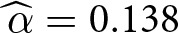 with a log-likelihood value of −8826.793 (Fig. 2) and predicted a true incidence of left-handedness 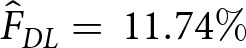 (eq. 2). These estimates are equal to those of Laland et al. up to three decimal places. See Table 1 for the predicted true incidence of each offspring type.

The goodness-of-fit of the model (with the MLE parameters) to the observed data was tested without adjustment (using matrix 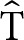). The full model with three parameters (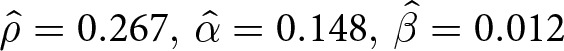) did not have a good fit for all studies combined (*G* = 556.73, *df* = 31, *p* < 10^−96^) and for only 8 of 17 studies individually (Table 3). The model with *β* fixed at zero (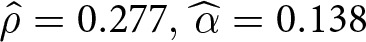) did not have a good fit for all studies combined (*G* = 556.03, *df* = 32, *p* < 10^−96^) and also for only 8 of 17 studies individually (Table 3). In most cases, our G statistics increased compared to those of Laland et al. These results do not match those presented by Laland et al., who report a good fit for 16 out of the 17 studies individually, as well as for all studies combined (Table 3, supplementary text S4).

**Table 3.** Comparison of goodness-of-fit results (G statistics) to Laland et al. (1995). See supplementary tables for more details

	Laland et al. (1995)	Scenario A		Scenario B		Scenario C	
	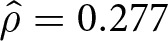	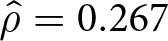	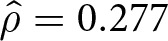	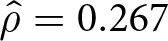	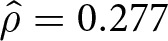	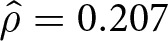	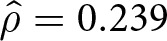
	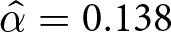	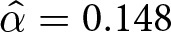	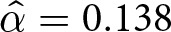	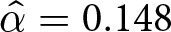	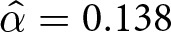	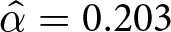	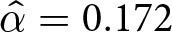
		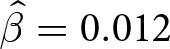		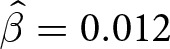		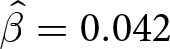	
Ramaley (1913)	13.19*	31.42*	31.72*	12.68*	13.19*	7.1*	8.29*
Chamberlain (1928)	5.09	237.87*	238.64*	4.65	5.1	3.31	4.22
Rife (1940)	4.37	4.16	4.6	3.91	4.37	2.77	3.79
Merrell (1957)	3.33	28.74*	28.26*	3.53	3.33	4.63	3.55
Annett (1973)	0.9	14.61*	14.57*	1.08	0.91	1.8	1.35
Ferronato et al. (1947)	0.54	0.77	0.77	0.56	0.54	0.48	0.4
Mascie-Taylor (unpub)^a^	0.11	3	3	0.11	0.11	0.2	0.19
Chaurasia & Goswani (unpub)^a^	2.27	20.25*	20.29*	2.12	2.27	2.11	2.65
Annett (1978)	3.26	8.6*	8.5*	3.43	3.26	3.59	3.04
Carter-Saltzmann (1980)	0.06	2.58	2.54	0.07	0.06	0.04	0.07
Coren & Porac (1980)	2.25	33*	33*	2.21	2.25	3.77	4.14
McGee and Cozad (1980)	3.8	156.43*	155.86*	3.81	3.8	0.63	0.24
McManus (1985) (ICM1)	0.01	2	1.98	0	0	0.06	0.09
McManus (1985) (ICM2prop)	2.61	3.53	3.39	2.7	2.61	2.33	1.98
McManus (1985) (ICM2mat)	0.88	1.35	1.35	0.88	0.88	0.83	0.81
McManus (1985) (ICM2pat)	0.01	3.01	3.03	0.01	0.01	0.01	0.01
Leiber & Axelrod (1981)	1.75	4.42	4.54	1.8	1.75	2.68	2.69
Overall	44.43	556.73*	556.03*	43.6	44.4	36.3	37.5

a These data are taken from McManus (1985).

* A significant difference between model and data at *p* = 0.05.

Thus, while our maximum-likelihood estimates closely match those of Laland et al., there were considerable differences in the goodness-of-fit test, suggesting that Laland et al. used the goodness-of-fit procedure with adjustment, which we validate in Scenario B.

### Scenario B: Estimating without adjustment, testing with adjustment

In this scenario, we estimate the model parameters without adjusting for criterion shift, as in scenario A, but test for goodness-of-fit with an adjustment, that is, using matrix *M* rather than *T*. Thus, parameter estimates are the same as in Scenario A (Fig. 2).

The full model with three estimated parameters provided a good fit for all studies combined (*G* = 43.6, *df* = 31, *p* = 0.067) and for 16 of 17 studies individually (Table 3, supplementary text S5). The model with two parameters and *β* fixed at zero produces similar results (all studies combined *G* = 44.4, *df* = 32, *p* = 0.071), which closely match those reported by Laland et al. (Table 3). The study in which the model has a poor fit is Ramaley (1913), as is the case in Laland et al. This study also showed the largest discrepancy in G statistic (Laland et al., *G* = 13.19; our analysis, *G* = 12.68).

The results of Scenario B closely match Laland et al.’s findings: maximum-likelihood estimates align to three decimal places, and goodness-of-fit tests yield similar results. Thus, Scenario B replicates Laland et al.’s analysis, suggesting they used maximum-likelihood estimation without adjustment and goodness-of-fit test with adjustment.

### Scenario C: Estimating and testing with adjustment

In this scenario, we estimate the model parameters and test the goodness-of-fit with an adjustment for criterion shift. The log-likelihood function in Laland et al. was denoted in terms of *measured incidence* of left-handers (*S_M_* in eq. 4), implying that they estimated the model parameters with adjustment. Hence, we sought to determine if Scenario C provides a better reproduction than Scenario B.

Estimating the parameters of the full model with adjustment resulted in a decrease in the estimated genetic transmission parameter, 
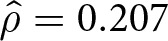
 (from 0.267) and an increase in the estimated cultural transmission parameters, 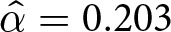 (from 0.148) and 
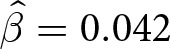
 (from 0.012), with a log-likelihood value of −8566.939 (Fig. 2). These parameter estimates give a higher *true incidence* of left-handedness, 
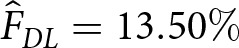
; see Table 1 for the predicted true incidence of each offspring type.

The model with two parameters and *β* fixed at zero shows a similar trend: a decrease in 
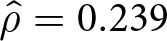
 (from 0.277) and an increase in 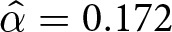 (from 0.138), with a log-likelihood value of −8567.529 (Fig. 2) and a similar higher *true incidence* of left-handedness, 
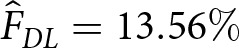
.

The full model with three parameters had a good fit for all studies combined (*G* = 36.3, *df* = 31, *p* = 0.234) and for 16 of 17 studies individually (Table 3). The model with two parameters had a good fit for all studies combined (*G* = 37.5, *df* = 32, *p* = 0.231), and for 16 of 17 studies individually (Table 3). As in Scenario B and in Laland et al., the one study the model did not fit was Ramaley (1913). However, the G statistics decreased for all combined studies and for 9 and 8 individual studies compared to Laland et al. in the three and two parameter models, respectively (Table 3, supplementary text S6).

Overall, maximum-likelihood estimation with the adjustment for criterion shift led to a decrease in the estimated *ρ* and an increase in the estimated *α* and *β*, an increase in the predicted *true incidence* of left-handedness, and a decrease in some of the *G* statistics compared to that reported by Laland et al. Thus, the results of Scenario C differ from those reported by Laland et al., reinforcing that Laland et al. did not apply the adjustment during estimation. Furthermore, these results suggest that adjusting for a criterion shift during both estimation and testing improves the goodness-of-fit of the model compared to adjusting just during testing.

### Goodness-of-fit test on twin data

Laland et al. (1995) also tested the goodness-of-fit of their model on twin data. We have reproduced this test using the MLE estimates from Scenario B with *β* fixed to zero and with adjustment for criterion shift.

Most expected frequencies and G statistics match those reported in Laland et al. (Table S10). Any discrepancies in the predicted expected values for the first 13 datasets are explained by using the rates of left-handedness reported by McManus (1985). Our calculated G statistic for all studies combined was slightly lower than reported by Laland et al. by 0.08, and our *p*-value was one half of that reported by Laland et al. Overall, this analysis provides a good reproduction of Laland et al.

### Evaluation of the estimation method

After confirming that Laland et al. estimated model parameters without adjustment as in Scenario B, we assess estimation performance using synthetic datasets generated with known parameter values (details on simulations in supplementary text S3) and compare it to estimation with adjustment as in Scenario C. We also attempted to improve the performance of Scenario B using a linear regression correction but only got minor improvement (supplementary text S7, Figure S4).

#### Estimation accuracy

**Figure 3.**
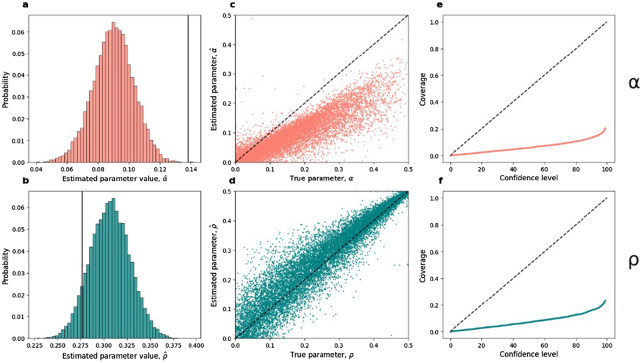
**Performance of the estimation method without adjustment on simulated synthetic data. (a, b)** The distribution of 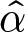 and 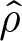 estimated from synthetic data simulated with the values estimated by Laland et al. (solid lines; *α* = 0.138 in panel a and *ρ* = 0.277 in panel b). **(c, d)** Scatter plot of parameter estimates (*y*-axis) vs. the true parameter (*x*-axis). **(e, f)** Coverage for various confidence levels: the rate at which the true parameter value falls within the confidence interval at a given confidence.

We evaluated the estimation accuracy for the MLE parameters reported by Laland et al., *ρ* = 0.277 and *α* = 0.138. We simulated 15,000 synthetic datasets with criterion shift and estimated the model parameters using the process in Scenario B. For each synthetic dataset, we took the estimate with the highest likelihood out of five runs of the Nelder–Mead algorithm with five initial guesses (*α* = *ρ* = 0.1, *α* = 0.1 and *ρ* = 0.01, *α* = 0.01 and *ρ* = 0.1, *α* = 0.01 and *ρ* = 0.45, and *α* = 0.45 and *ρ* = 0.01). The distribution of the 15,000 estimated parameter sets indicates that *α* is underestimated with a bias of 0.048 and *ρ* is overestimated with a bias of 0.029 (Fig. 3a and 3b).

We then simulated 15,000 additional synthetic datasets with a criterion shift to determine if these biases are general. For each simulation, *ρ* was sampled uniformly from 0 to 0.5, and *α* was sampled uniformly from 0 to 0.5-*ρ*. We then simulated the synthetic datasets with a criterion shift and estimated the parameters from the synthetic data. The mean squared error between the true and estimated parameter value is 0.0068 and 0.0025 for *α* and *ρ*, respectively. Overall, estimation without adjustment frequently underestimates *α* and overestimates *ρ* (Fig. 3c and 3d). Thus, the bias is consistent across parameter values.

#### Confidence interval and coverage

We measured the estimation method’s true coverage, defined as the rate at which the true parameter is contained within the estimated parameter confidence interval (CI); that is, for a CI with a confidence level of *c*%, the true parameter is expected to be contained within the CI in *c*% of the simulated synthetic datasets (Schall, 2012).

We used the 15,000 synthetic datasets with criterion shift and uniformly sampled parameters from above. We used non-parametric bootstrap with 200 resamples to compute the CI, generating 200 parameter estimates per synthetic dataset. We then calculated CIs for various confidence levels from these 200 estimates. Finally, the fraction of 15,000 synthetic datasets in which the true parameter is within the CI was computed for each confidence level (Fig. 3e and 3f). For both model parameters, the true coverage was significantly below the intended rate; for the standard confidence level of 95%, less than 20% of the 15,000 CIs contained the true parameter values.

#### Estimation with adjustment

We repeated the above evaluation for parameter estimation with adjustment, as in Scenario C. Estimation of *α* improved compared to Scenario B, while estimation of *ρ* did not (mean squared error for *α* and *ρ* is 0.002 and 0.01, respectively; Figures S5a and S5b). We observed that most estimation errors occur when the true *α* is low or when the true *α + ρ* is close to 0.5, so that when we filter out simulations with *α* < 0.02 or *ρ + α* > 0.49, the mean squared error for *α* and *ρ* drops to 0.0005 and 0.001, respectively. Indeed, when *α* is low, or α + ρ is close to 0.5, then the frequency of right-handed offspring is roughly ½ + *ρ* or 1 in all mating types, respectively. This situation leads to practical non-identifiability, meaning that the data contain too little information to distinguish among parameters values and hence to estimate them reliably.

When estimating *α* and *ρ* from real data, however, we do not know if *α* is low or if *α + ρ* is close to 0.5. Therefore, we determine these cases from the data. For low *α*, we expect the frequency of right-handed offspring to be very similar across mating types. For *α + ρ* close to 0.5, we expect an overall low frequency of left-handedness. Thus, we filtered datasets under either of these conditions: (i) when the frequency of left-handedness is below 1% in either the parent or offspring generation and (ii) if the difference between the frequency of right-handed offspring of two right-handed parents (*p*(*R|R × R*)) and of two left-handed parents (*p*(*R|L × L*)) is less than 7%. Importantly, these two cases do not occur in the datasets reported by Laland et al. and are unlikely to be observed in real datasets. Estimation under these conditions is much more accurate (mean squared error for *α* and *ρ* is 0.0003 and 0.001, respectively; Figures S5c and S5d), and the true coverage is well-aligned with the intended rate (Figures S5e and S5f). We therefore conclude that estimation with adjustment (Scenario C) should be used, except when the above conditions (i-ii) do not apply.

### Sex differences in handedness

We tested for sex differences in hand preference determination. We extended Laland et al.’s model to include sex differences in parental effects and offspring response (Methods, Table S11). As in Laland et al., we assumed fixation of the *D* allele and estimated the model parameters using a maximum-likelihood approach after adjusting for criterion shift (Scenario C).

We fitted five models, which differ by the number of free parameters, to the McKeever familial dataset using the log-likelihood function of the extended model, S* (eq. 6). Parameter estimates are in Table S12. Likelihood ratio tests comparing nested models revealed statistically significant differences in model fit (all *p*-values < 10^−5^ except for Laland et al.’s two- and three-parameter models, for which *p* = 0.07), indicating that the models accounting for sex differences provide a significantly better fit to the data, but not directly contradicting Laland et al.’s hypothesis that *β* can be omitted (see Table 4 for likelihood ratio test results.) Thus, the data supports model V, which has a total of seven parameters, *ρ, γ_F_, γ_M,_ β_F_, β_M,_ α_F_*, and *α_M_*. We conclude that there are significant sex differences in the transmission of hand preference at both the parental and offspring levels.

**Table 4.** Results of likelihood ratio tests comparing nested models with sex differences for the McKeever (2000) dataset, and Generation 1 and Generation 2 data from Nurhayu et al. (2020)

odel	# Param.	*df*	*McKeever LR*	*p*-Value	*Generation 1 LR*	*p*-Value	*Generation 2 LR*	*p*-Value
I vs. II	2/3	1	3.36	0.07	0.18	0.67	1.66	0.07
II vs. III	3/4	1	24.50	7.4 × 10^−7^	3.24	0.07	0.22	0.64
II vs. IV	3/5	2	26.92	1.4 × 10^−6^	21.76	1.9 × 10^−5^	23.50	7.0 × 10^−6^
III vs. V	4/7	3	28.72	2.6 × 10^−6^	23.32	3.5 × 10^−5^	23.38	3.4 × 10^−5^
II vs. V	3/7	4	53.22	7.7 × 10^−11^	26.56	2.4 × 10^−5^	23.60	9.6 × 10^−5^

For each pair, the number of parameters (# Param.), Test statistic (LR), degrees of freedom (*df*), and *p*-value are reported. Significant *p*-values (*p* < 0.05) indicate an improved fit of the more complex model

We then fitted the same five models to two additional datasets, for two generations in Indonesian populations (Nurhayu et al., 2020). We found significant difference in model fit only for models II vs. IV, II vs. V, and III vs. V (*p < 0.05*; Table 4). Thus, while the data supports model V, these results suggest that the significant improvement in model V is due to including sex differences in offspring handedness rather than including a non-neutral effect of having one right-handed and one left-handed parent. We conclude that there are significant sex differences in offspring handedness transmission.

## Discussion

We reproduced Laland et al.’s results and found that adjusting for criterion shift during both estimation and testing (Scenario C) yields more accurate estimates, better fit, a larger cultural component, and higher expected left-handed prevalence than adjusting only during testing (Scenario B). Our extensions with sex differences suggest stronger maternal than paternal effects and stronger effects on daughters than sons. We now turn to a detailed discussion of these results.

To reproduce the analysis conducted by Laland et al. (1995), we examined three analytic scenarios (Fig. 1). In scenario A, we performed maximum-likelihood estimation and goodness-of-fit test without adjusting for criterion shift. The parameter estimates closely matched those reported by Laland et al., but the goodness-of-fit test results were incompatible with those of Laland et al. In scenario B, we performed the estimation without adjustment but adjusted model predictions for criterion shift before testing for goodness-of-fit. This produced a good reproduction of Laland et al. In scenario C, we performed the estimation and the goodness-of-fit test with adjustment for criterion shift. The estimates differed from those of Laland et al., leading to differences in the goodness-of-fit statistics. Therefore, Scenario B gave the best reproduction of Laland et al. Furthermore, we tested the model with the estimated parameters from Scenario B (with *β* fixed at zero) on twin data (separate from the familial data used for parameter estimation), which again reproduced the results of Laland et al.

We find that Laland et al.’s estimation method (Scenario B) shows systematic bias and CI misalignment. When evaluating it on synthetic data simulated from known parameter values, it consistently overestimates *ρ* and underestimates *α* and produces too-wide CIs that do not contain the true parameter value. However, adjusting for criterion shift (Scenario C), parameters are successfully estimated, and adequate CIs are produced. Thus, future applications should consider using the estimation method of Scenario C.

The criterion shift had notable implications: adjusting for the shift during parameter estimation increased the estimated effect of cultural versus genetic transmission and the overall expected prevalence of left-handers in the population. Adjusting during goodness-of-fit testing improved the test results when estimating without adjustment (Scenario B) and even more so when estimating with adjustment (Scenario C). Moreover, estimation without adjustment, as used in Laland et al., was biased and underconfident: it consistently overestimated *ρ*, underestimated *α*, and produces CIs that do not contain the true parameter value (Fig. 3). In contrast, estimation with adjustment was accurate with good coverage (Figure S5). We note that the adjustment introduces non-linear constraints on the model parameters (through *M* and *P*) and may create curves in the log-likelihood surface in regions where it is undefined (Figure S6). Such irregularities can cause convergence problems for the algorithm that maximizes the log-likelihood.

When estimating the parameters of the full model, *ρ, α,* and *β*, Laland et al. estimated *β* close to zero, and therefore used a two-parameter model in which *β* was fixed at zero, which implies that the contrasting cultural effect of mixed-handed parents cancels out (Table 2). We found that fixing 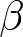 at zero increased the estimated genetic transmission *ρ* by 3.7% and 15.4% without and with the adjustment, respectively, and decreased the value of same-parent cultural transmission *α* by 6.75% and 15.27% without and with the adjustment, respectively. Estimates of *β* were smaller than the other parameters and close to zero in all scenarios. The log-likelihood was slightly higher when estimating *β*, which is expected in a model with an additional parameter, and the G statistic across all studies combined decreased. We therefore performed a likelihood-ratio test between the two versions of the model and found that the evidence does not support estimating *β* from the data (*F*(*1,14) = 0.00024, p = 0.99*). Therefore, fixing it at zero appears to be a reasonable simplification, suggesting that, at least in this dataset, the cultural effect of two parents with opposite hand preferences cancels out.

### Implications

A large body of literature suggests the combined influences of genes and culture on human handedness. Handedness thus appears well suited to a gene-culture co-evolutionary analysis. However, Laland et al. is to date the only study that has attempted such an analysis. Since its publication in 1995, their model and analysis have yet to be reproduced, extended, or applied to new data. The process in Scenario B enabled the successful reproduction of their analysis, and the process in Scenario C provided accurate estimates with good fit to the data and good coverage (i.e., correct CIs). We therefore provide an updated open-source implementation of their analysis in the Python programming language, see https://github.com/yoavram-lab/Laland1995. We further demonstrate how their model can be extended to test hypotheses on cultural transmission of handedness. Our extended analysis can be applied to other studies of gene-culture co-evolution.

### Sex differences in handedness

The model of Laland et al. (1995) does not account for sex differences in the transmission of handedness preference. The parameter *β*, when included in the three-parameter model, accounts for the influence of parents with mixed-handedness but not for the sex of the right-handed parent. Indeed, maternal and paternal effects may not be equal, and differential parental influence has been debated in the literature (e.g., Annett, 1999; McGee & Cozad, 1980; McKeever, 2000). We therefore extended their model to include sex-differences in both the parental and offspring generation. We fit the extended models I-V on the McKeever dataset; therefore, parameter estimates differ from those of Laland et al. We also fit the same models on the datasets Generation 1 and Generation 2 from Nurhayu et al. (2020).

In fitting the extended models with sex differences, we did not constrain *β* or *γ* to be positive. A negative value for *β* or *γ* indicates a cultural bias towards left-handedness whereas a positive value indicates a bias towards right-handedness. In model III, which includes parental sex differences, the estimated parental effects for the McKeever dataset are *β_F_ = β_M_* = −0.0159 and *γ_F_ = γ*_M_ = −0.0918. The average effect is, therefore, −0.0539, which is very close to the estimate in model II (without sex differences) of *β* = −0.0482.

In considering sex differences in parental effects, we find that cultural maternal effects on handedness are stronger than paternal, that is, *γ < β* (McKeever model III: *γ_F_* = *γ_M_* = −0.0918 < −0.0159 = *β_F_* = *β_M_*; Gen. 1 model III, *γ_F_* = *γ_M_* = −0.1335 < −0.0121 = *β_F_* = *β_M_*, Gen. 2 model III, *γ_F_* = *γ_M_* = −0.2488 < −0.2031 = *β_F_* = *β_M_).* In models where we also have differences in the inheritance of heritability due to sex of offspring, this result holds across sexes for the McKeever dataset (model V: *β_F_* > *β_M_* > *γ_F_* > *γ_M_*; Table S12), and between sexes for the Nurhayu et al. data, (Gen. 1, Gen. 2 model V: *β_F_* > *γ_F_* > *β_M_* > *γ_M_*; Tables S13 and S14). That is, the effect of mothers on offspring handedness is stronger in both datasets, as is the sensitivity of daughters. Similar results have been described before in humans (McGee & Cozad, 1980) and non-human animals (Zefferman 2016). Maternal effects could be stronger because mothers spend more time than fathers with their children, e.g., practicing writing skills (Morgan et al., 2009). When considering offspring sex differences in all three datasets, we find that daughters are more strongly affected than sons by same-handed parents, that is, *α_F_* > *α_M_* (e.g., for the McKeever data, models IV and V: *α_F_* = 0.0335, *α_M_* = 0.0163). Indeed, studies suggest that female offspring are more likely to switch from left-handed to right-handed (Coren & Halpern, 1991).

The individuals sampled by Nurhayu et al. (2020) are from agricultural populations in Indonesia, whereas the individuals studied by McKeever et al. (2000) were primarily college students from the UK and US. Although we analyse the two generations from Nurhayu et al. (2020) as two datasets under the assumption that the parameters may differ, as culture may change between generations, we find similar trends in the model parameter estimated values and the model ranking. Comparing the parameter estimates to those of the McKeever dataset, we find that the effect of mothers is strong in the UK and US college students, whereas the sensitivity of daughters is strong in the Indonesian population (*β_F_* > *γ_F_* > *β_M_* > *γ_M_* for Nurhayu et al. (2020) but *β_F_* > *β_M_* > *γ_F_* > *γ_M_* for the McKeever dataset). It is not surprising to find differences in parameter estimates between different cultures.

#### Future directions

Our study provides an accurate, updated, and accessible implementation of the original analysis of Laland et al. (1995). The specific parameter estimates and other results depend on the cultural context in which the data were collected. Using the updated analysis presented here, future research could study data from different cultures and time periods, possibly revealing different patterns of transmission of handedness.

Future work can compare the model in this study to other influential models in the field, e.g., McManus (1985) and Annett (1972). These models were revised by their authors in the decades following their publication (Annett, 2002; McManus & Bryden, 1992), but updated analysis protocols were not published. Given the difficulties we encountered in reproducing Laland et al., it is possible that similar reproducibility gaps also exist in other studies. Reproducing these studies could facilitate an empirical comparison of different models. This will constitute a milestone in the ongoing debate on the origins of human handedness.

## Supporting information

Karstadt et al. supplementary material Karstadt et al. supplementary material

## Supplementary material

The supplementary material for this article can be found at https://doi.org/10.1017/ehs.2026.10038.

## Research transparency and reproducibility interest

The authors have made the code used to produce these results available. No new data collected in this study. Data used for analysis is cited and appears in tables and supplementary tables. ChatGPT was used for editing specific sections of the manuscript.
