# Supplementary material for: Transmission of human handedness: a reanalysis
Source: Evol Hum Sci. 2026 Feb 16;8:e8. doi: 10.1017/ehs.2026.10038 (PMC12964082; doi:10.1017/ehs.2026.10038)
Supplement: Karstadt et al. supplementary material [file S2513843X26100383sup001.docx]

**Supplementary information**

**Transmission of human handedness: a reanalysis**

Rony Karstadt, Chloe Shiff, Tomer Oron, Nadav Ben Nun, Yoav Ram

28 October 2025

# Supplementary Text

S1: Methods - Familial data

The data used in this study (Table S3) are the same as in Table 3 of Laland et al. (1995). None of the datasets were excluded, and the data were not transformed. The data combine 17 earlier studies published between 1913 and 1985. To ascertain the properties of this dataset, we examined the data in the original studies. We used Table 3 in Nurhayu et al. (2019), which describes the properties of these studies, except for Ferronato et al. (1947), in which the properties of the sample are not detailed. The remaining 16 studies sampled US and UK populations between 1911 and 1980, of which 14 studies focused on college students and staff and two studies focused on the general population: Mascie-Taylor (unpublished, data in McManus 1985) and Carter-Saltzmann (1980). Handedness measurements and criteria vary among the studies, including self-reporting, hand used for writing, and questionnaires. Consequently, all 17 studies present data on hand preference (rather than hand performance).

In the familial data presented by Laland et al., the frequency of left-handers varies across generations and studies (Figure S1). In 15 of the 17 studies, the frequency of left-handers increased between parent and offspring generations, with the exceptions of Ferronato et al. (1947), in which the demography of subjects and criteria for left-handedness are not described, and Mascie-Taylor (unpublished), in which the participants were sampled from the general UK population, and the criteria for left-handedness are not described.

The highest frequency of left-handers is in McGee & Cozad (1980) for both the parent (18.02%) and offspring (24.57%) generation. This sample focused on US college students, and their criteria for left-handed subjects was using the left hand in at least one out of 10 tasks. The lowest frequency of left-handers is reported in Chamberlain (1928) for both parent generation (3.56%) and offspring generation (4.77%). This sample focused on US students and used the writing hand as the criterion for a left- or right-handed subject. Within parental mating, left-handers constitute 8.5% of the offspring of two right-handed parents; 22.1% of mixed-handed parents, and 38.1% of two left-handed parents (Table 3 and Figure S1).

We found differences between the data reported in the original studies and in Laland et al. (1995). These differences appear to stem from reliance on secondary data sources without direct validation. However, organizing the data in the field is beyond the scope of the present study, so we use the data reported by Laland et al.

S2: Methods - Degrees of freedom for G-test

According to Laland et al. (1995, p. 444): “Since for each study estimates of m_p_ and m_o_ are computed from the data, and two parameters of the model are estimated across the entire data set (α and ρ), the appropriate number of degrees of freedom per study is 32/17 = 1.88.” However, they did not explicitly report the degrees of freedom for a model with three parameters (when β is inferred rather than fixed). We deduced that as each study in the data is a 3x2 table of offspring handedness given parental handedness, it contributes (3-1) (2-1)=2 degrees of freedom. There are 17 studies, and therefore, the entire dataset comprises 2x17=34 degrees of freedom. The number of parameters estimated across the entire dataset should be subtracted from this overall number of degrees of freedom. Thus, when estimating ρ and α, the overall number of degrees of freedom is 34-2=32, and when estimating ρ, α, and β the overall number of degrees of freedom is 34-3=31. The final number of degrees of freedom per study is 32/17=1.88 when estimating ρ and α, and 31/17=1.82 when estimating ρ, α and β.

S3: Methods - Simulations

We simulate synthetic population data using Laland et al.'s model and given values for the model parameters *ρ*, *α*, and *β*. The synthetic data contains 17 datasets presenting triplet as in Table S3. As the number of families in each dataset varies, we draw the number of families from the uniform distribution *U(100, 2,200)*. The number of offspring per family is drawn from the multiple offspring datasets of McManus (1985) as *1+Bin (4, 0.354)*. The mating types are drawn from *Bin(2, F_DL_ )*, where *F_DL_* is computed from the model parameters (eqs. 1-2). Then, the number of left-handed offspring per family is drawn from a multinomial distribution with offspring probabilities conditioned on mating type as in Table S1.

We simulate a criterion shift by choosing two observed handedness rates from those reported in the datasets in Laland et al.: one for the parent's generation *m_p_*, and one for the offspring's generation, *m_o_*. The chosen rates are then scaled for each dataset by multiplying with the true rate of left-handedness and dividing by Laland et al.'s reported true rate. The matrices *O* and *P* are then computed, and the synthetic number of mating types and number of left-handed offspring are drawn using the conditional probabilities in *O* and *P*.

S4: Results – Scenario A goodness-of-fit test

We did not replicate the same G statistics as Laland et al. for any of the 17 studies. The most notable disparity in G statistics is that in the data from Chamberlain (1928): in Laland et al., G=5.09, whereas our analysis yielded G=237.87 for a model with three parameters and G=238.64 for a model with two parameters. The smallest difference occurred in the data of Rife (1940), where G=4.37 in Laland et al. and G=4.16 and G=4.6 in our analysis for models with three and two parameters, respectively. The latter was the only G statistic in our analysis smaller than Laland et al. Overall, our analysis produced G statistics that differed from those reported by Laland et al. for all 17 studies. Additionally, when combining all studies, the G statistic increased from 44.43 in the original analysis to 556.73 and 556.03 for the models with three and two parameters, respectively.

S5: Results – Scenario B goodness-of-fit test

The model with three parameters produced the same G statistic as Laland et al. in 4 of 17 studies. Of the 16 studies with a good fit, the largest discrepancy was the study by Rife (1940), with G=4.37 in Laland et al. and G=3.91 in our analysis. When fitting across all studies combined, the G statistic in our analysis was 43.6, slightly lower than that of Laland et al., with G=44.43.

The model with two parameters produced the same G statistic as Laland et al. in 14 of 17 studies. The discrepancy in G statistics for the remaining three studies was smaller than 0.01. When fitting across all studies combined, the G statistic in our analysis was 44.4, identical to Laland et al. The differences between our results and those of Laland et al. likely reflect numerical errors.

S6: Results – Scenario C goodness-of-fit test

Among all 17 studies, the biggest discrepancy in G statistics was in Ramaley (1913) for both sets of estimates: while Laland et al. reported G=13.19, the model with three parameters produced G=7.1, and the model with two parameters produced G=8.29. Furthermore, out of the 16 studies with a good fit, the biggest discrepancy in G statistics was in the study of McGee & Cozad (1980): G=3.80 in Laland et al. versus G=0.63 and G=0.24 in our analysis for the model with three and two parameters, respectively. We obtained the same G statistic as Laland et al. for just one study, McManus (1985) (ICM2pat), G=0.01. When fitting across all studies combined, our analysis produced a G statistic lower than that of Laland et al., G=36.3 (three parameters) and G=37.5 (two parameters) compared to G=44.43.

S7: Results – linear regression correction

To correct the estimation bias, we used linear regression to predict true parameters from estimated parameters. The linear regression was trained on the 15,000 parameter values estimated from synthetic data, for which we know the true parameter values. The linear regression provided a good fit and slightly improved the true coverage, but it was still far lower than the intended rate (mean squared error for *α* and *ρ* is 0.0021 and 0.0014, respectively; Figure S4). Applying this correction to Laland et al.'s estimates changed the estimated values: *α*=0.223 and *ρ*=0.229 rather than *ρ*=0.277 and *α*=0.138. These “corrected” estimates had a good fit only for three of the datasets and they did not fit the data for all studies combined (*G* = 1210.01, *p*<0.001), indicating that the estimation method of Scenario B still fails to estimate the true parameters.

S8: Methods – Extended model with sex differences

Here, we provide the recursions of the extended model with sex differences, used to compute the equilibrium handedness frequencies. $F_{XY}^{F}$ and $F_{XY}^{M}$ represent the frequencies in the current generation of allele *X* (either *D* or *C*) in *Y*-handed female and male individuals, respectively, (*Y*=*R* for right-handed and *Y=L* for left-handed individuals). $F_{XY}^{'}$ represents the frequencies in the next generation. The equations are written once without sex as $F_{XY}^{'}$ for simplicity, but are computed twice, for male frequencies $F_{XY}^{M^{'}}$ and for female frequencies, $F_{XY}^{F^{'}}$. (1995). $P_{X\times Y,ZQ}$ is the probability of an individual being right-handed (1) given a *ZQ* genotype, (2) the mother is *X*-handed, and (3) the father is *Y*-handed, see Table S11. We set $h_{1}=0,h_{2}=0,s_{1}=0,s_{2}>0$, as in Laland et al. (1995)

# Supplementary Tables

**Table S1.** The probability of right-handed offspring with genotype DD, DC, and CC given RxR, RxL, and LxL parental phenotypes. Genetic effects are determined by parameters *ρ* (dexterity) and *h_1_* (allele dominance). Cultural effects are determined by *α* (same-hand parental effect) and *β* (mixed-hand parental effect).

| Parental mating | Offspring genotype | | |
| --- | --- | --- | --- |
|  | DD | DC | CC |
| Right X Right | $\frac{1}{2}+\rho+\alpha$ | $\frac{1}{2}+h_{1}\rho+\alpha$ | $\frac{1}{2}+\alpha$ |
| Right X Left | $\frac{1}{2}+\rho+\beta$ | $\frac{1}{2}+h_{1}\rho-\beta$ | $\frac{1}{2}+\beta$ |
| Left X Left | $\frac{1}{2}+\rho-\alpha$ | $\frac{1}{2}+h_{1}\rho-\alpha$ | $\frac{1}{2}-\alpha$ |

**Table S2.** The expected *true incidence* of right-right, right-left, and left-left-handed twins given parental phenotypes and assuming the D allele is fixed in the population.

| Parental mating |  | Twins Phenotypes | |
| --- | --- | --- | --- |
|  | R-R | R-L | L-L |
| Right x Right | $\left( \frac{1}{2}+\rho+\alpha\right)^{2}$ | $2\left( \frac{1}{2}-\rho-\alpha\right)\left( \frac{1}{2}+\rho+\alpha\right)$ | $\left( \frac{1}{2}-\rho-\alpha\right)^{2}$ |
| Right x Left | $\left( \frac{1}{2}+\rho+\beta\right)^{2}$ | $2\left( \frac{1}{2}-\rho-\beta\right)\left( \frac{1}{2}+\rho+\beta\right)$ | $\left( \frac{1}{2}-\rho-\beta\right)^{2}$ |
| Left x Left | $\left( \frac{1}{2}+\rho-\alpha\right)^{2}$ | $2\left( \frac{1}{2}-\rho+\alpha\right)\left( \frac{1}{2}+\rho-\alpha\right)$ | $\left( \frac{1}{2}-\rho+\alpha\right)^{2}$ |

**Table S3. Data and results reported by Laland et al. (1995).** The frequency of left- and right-handed offspring born to different parental phenotypes in 17 studies: two right-handed parents (Right x Right), mixed-handed parents (Right x Left), and two left-handed parents (Left x Left). The model predictions from Laland et al. are shown in parentheses for parameter estimates $\hat{\rho}=0.277,\hat{\alpha}=0.138$, and $\hat{\beta}=0$. With these predictions, the model fits the data in 16 of 17 studies and across all studies combined (G= 44.33, df= 32, p>0.05).

| Parental mating | Right x Right | | Right x Left | | Left x Left | |  |
| --- | --- | --- | --- | --- | --- | --- | --- |
| Offspring | Right | Left | Right | Left | Right | Left | G_df=1.82_ |
| Ramaley (1913) | 841 (874.7) | 115 (81.3) | 113 (130.1) | 54 (36.9) | 1 (5.0) | 7 (3.0) | 31.42* |
| Chamberlain (1928) | 6917 (6610.9) | 308 (614.1) | 411 (361.5) | 53 (102.5) | 18 (15.5) | 7 (9.5) | 237.87* |
| Rife (1940) | 1842 (1823.6) | 151 (169.4) | 140 (135.5) | 34 (38.5) | 5 (6.8) | 6 (4.2) | 4.16 |
| Merrell (1957) | 140 (159.2) | 34 (14.8) | 33 (41.3) | 20 (11.7) | 8 (6.2) | 2 (3.8) | 28.74* |
| Annett (1973) | 6206 (6290.6) | 669 (584.4) | 471 (464.3) | 125 (131.7) | 5 (3.7) | 1 (2.3) | 14.61* |
| Ferronato et al. (1947) | 154 (151.0) | 11 (14.0) | 31 (31.2) | 9 (8.8) | 0 (0.0) | 0 (0.0) | 0.77 |
| Mascie-Taylor (unpub)^b^ | 232 (227.8) | 17 (21.2) | 41 (37.4) | 7 (10.6) | 3 (2.5) | 1 (1.5) | 3 |
| Chaurasia & Goswani (unpub)^b^ | 1060 (1101.7) | 144 (102.3) | 122 (130.9) | 46 (37.1) | 3 (4.3) | 4 (2.7) | 20.25* |
| Annett (1978) | 1656 (1634.2) | 130 (151.8) | 170 (163.6) | 40 (46.4) | 4 (2.5) | 0 (1.5) | 8.6* |
| Carter-Saltzmann (1980) | 303 (311.1) | 37 (28.9) | 45 (46.7) | 15 (13.3) | 0 (0.0) | 0 (0.0) | 2.58 |
| Coren & Porac (1980) | 315 (350.4) | 68 (32.6) | 57 (56.9) | 16 (16.1) | 0 (0.0) | 0 (0.0) | 33* |
| McGee & Cozad (1980) | 848 (969.0) | 211 (90.0) | 325 (370.0) | 150 (105.0) | 30 (32.2) | 22 (19.8) | 156.43* |
| McManus (1985) (ICM1) | 58 (61.3) | 9 (5.7) | 14 (14.8) | 5 (4.2) | 0 (0.0) | 0 (0.0) | 2 |
| McManus (1985) (ICM2prop) | 134 (136.3) | 15 (12.7) | 17 (20.3) | 9 (5.7) | 1 (0.6) | 0 (0.4) | 3.53 |
| McManus (1985) (ICM2mat) | 74 (71.4) | 4 (6.6) | 6 (6.2) | 2 (1.8) | 0 (0.0) | 0 (0.0) | 1.35 |
| McManus (1985) (ICM2pat) | 86 (82.3) | 4 (7.7) | 8 (7.0) | 1 (2.0) | 0 (0.0) | 0 (0.0) | 3.01 |
| Leiber & Axelrod (1981) | 1729 (1740.3) | 173 (161.7) | 281 (268.0) | 63 (76.0) | 8 (6.8) | 3 (4.2) | 4.42 |
| ^b^ These data are taken from McManus (1985). | | | | | | | |
| * A significant difference between model and data at p = 0.05. | | | | | | | |

**Table S4. Scenario A: *Results of g*oodness-of-fit test without adjustment for estimates without adjustment *with three parameters*** $\hat{\boldsymbol{\rho}}$**= 0.267,** $\hat{\boldsymbol{\alpha}}$ **= 0.148,** $\hat{\boldsymbol{\beta}}$**= 0.012.** Model predictions are in parentheses, and G *statistics* for each study are in the right column. Model predictions fit the data in 9 of 17 individual studies and do not fit for all studies combined (G=556.73, df=31, p<0.05).

| Parental mating | Right x Right | | Right x Left | | Left x Left | |  |
| --- | --- | --- | --- | --- | --- | --- | --- |
| Offspring | Right | Left | Right | Left | Right | Left | G_df=1.88_ |
| Ramaley (1913) | 841 (874.7) | 115 (81.3) | 113 (129.8) | 54 (37.2) | 1 (5.1) | 7 (2.9) | 31.72* |
| Chamberlain (1928) | 6917 (6610.9) | 308 (614.1) | 411 (360.5) | 53 (103.5) | 18 (16.0) | 7 (9.0) | 238.64* |
| Rife (1940) | 1842 (1823.6) | 151 (169.4) | 140 (135.2) | 34 (38.8) | 5 (7.0) | 6 (4.0) | 4.6 |
| Merrell (1957) | 140 (159.2) | 34 (14.8) | 33 (41.2) | 20 (11.8) | 8 (6.4) | 2 (3.6) | 28.26* |
| Annett (1973) | 6206 (6290.6) | 669 (584.4) | 471 (463.1) | 125 (132.9) | 5 (3.8) | 1 (2.2) | 14.57* |
| Ferronato et al. (1947) | 154 (151.0) | 11 (14.0) | 31 (31.1) | 9 (8.9) | 0 (0.0) | 0 (0.0) | 0.77 |
| Mascie-Taylor (unpub)^b^ | 232 (227.8) | 17 (21.2) | 41 (37.3) | 7 (10.7) | 3 (2.6) | 1 (1.4) | 3 |
| Chaurasia & Goswani (unpub)^b^ | 1060 (1101.7) | 144 (102.3) | 122 (130.5) | 46 (37.5) | 3 (4.5) | 4 (2.5) | 20.29* |
| Annett (1978) | 1656 (1634.2) | 130 (151.8) | 170 (163.2) | 40 (46.8) | 4 (2.6) | 0 (1.4) | 8.5* |
| Carter-Saltzmann (1980) | 303 (311.1) | 37 (28.9) | 45 (46.6) | 15 (13.4) | 0 (0.0) | 0 (0.0) | 2.54 |
| Coren & Porac (1980) | 315 (350.4) | 68 (32.6) | 57 (56.7) | 16 (16.3) | 0 (0.0) | 0 (0.0) | 33* |
| McGee & Cozad (1980) | 848 (969.0) | 211 (90.0) | 325 (369.1) | 150 (105.9) | 30 (33.2) | 22 (18.8) | 155.86* |
| McManus (1985) (ICM1) | 58 (61.3) | 9 (5.7) | 14 (14.8) | 5 (4.2) | 0 (0.0) | 0 (0.0) | 1.98 |
| McManus (1985) (ICM2prop) | 134 (136.3) | 15 (12.7) | 17 (20.2) | 9 (5.8) | 1 (0.6) | 0 (0.4) | 3.39 |
| McManus (1985) (ICM2mat) | 74 (71.4) | 4 (6.6) | 6 (6.2) | 2 (1.8) | 0 (0.0) | 0 (0.0) | 1.35 |
| McManus (1985) (ICM2pat) | 86 (82.3) | 4 (7.7) | 8 (7.0) | 1 (2.0) | 0 (0.0) | 0 (0.0) | 3.03 |
| Leiber & Axelrod (1981) | 1729 (1740.3) | 173 (161.7) | 281 (267.3) | 63 (76.7) | 8 (7.0) | 3 (4.0) | 4.54 |
| ^b^ These data are taken from McManus (1985). | | | | | | | |
| * A significant difference between model and data at p = 0.05. | | | | | | | |

**Table S5. Scenario A: Results of goodness-of-fit test without adjustment for estimates without adjustment with two parameters** $\hat{\boldsymbol{\rho}}$ **= 0.277,** $\hat{\boldsymbol{\alpha}}$ **= 0.138.** Model predictions are in parentheses, and G statistics for each study are in the right column. Model predictions fit the data in 9 of 17 individual studies and do not fit for all studies combined (G=556.03, df=32, p<0.05).

| Parental mating | Right x Right | | Right x Left | | Left x Left | |  |
| --- | --- | --- | --- | --- | --- | --- | --- |
| Offspring | Right | Left | Right | Left | Right | Left | G_df=1.82_ |
| Ramaley (1913) | 841 (826.7) | 115 (129.3) | 113 (123.4) | 54 (43.6) | 1 (4.7) | 7 (3.3) | 12.68* |
| Chamberlain (1928) | 6917 (6906.9) | 308 (318.1) | 411 (419.7) | 53 (44.3) | 18 (21.1) | 7 (3.9) | 4.65 |
| Rife (1940) | 1842 (1838.3) | 151 (154.7) | 140 (143.8) | 34 (30.2) | 5 (7.9) | 6 (3.1) | 3.91 |
| Merrell (1957) | 140 (137.8) | 34 (36.2) | 33 (37.3) | 20 (15.7) | 8 (6.0) | 2 (4.0) | 3.53 |
| Annett (1973) | 6206 (6214.3) | 669 (660.7) | 471 (469.9) | 125 (126.1) | 5 (3.9) | 1 (2.1) | 1.08 |
| Ferronato et al. (1947) | 154 (152.5) | 11 (12.5) | 31 (32.5) | 9 (7.5) | 0 (0.0) | 0 (0.0) | 0.56 |
| Mascie-Taylor (unpub)^b^ | 232 (232.7) | 17 (16.3) | 41 (40.3) | 7 (7.7) | 3 (2.9) | 1 (1.1) | 0.11 |
| Chaurasia & Goswani (unpub)^b^ | 1060 (1067.6) | 144 (136.4) | 122 (127.0) | 46 (41.0) | 3 (4.2) | 4 (2.8) | 2.12 |
| Annett (1978) | 1656 (1652.5) | 130 (133.5) | 170 (174.7) | 40 (35.3) | 4 (2.9) | 0 (1.1) | 3.43 |
| Carter-Saltzmann (1980) | 303 (301.8) | 37 (38.2) | 45 (45.5) | 15 (14.5) | 0 (0.0) | 0 (0.0) | 0.07 |
| Coren & Porac (1980) | 315 (320.5) | 68 (62.5) | 57 (52.2) | 16 (20.8) | 0 (0.0) | 0 (0.0) | 2.21 |
| McGee & Cozad (1980) | 848 (828.0) | 211 (231.0) | 325 (335.5) | 150 (139.5) | 30 (32.3) | 22 (19.7) | 3.81 |
| McManus (1985) (ICM1) | 58 (58.0) | 9 (9.0) | 14 (14.0) | 5 (5.0) | 0 (0.0) | 0 (0.0) | 0 |
| McManus (1985) (ICM2prop) | 134 (131.7) | 15 (17.3) | 17 (19.6) | 9 (6.4) | 1 (0.6) | 0 (0.4) | 2.7 |
| McManus (1985) (ICM2mat) | 74 (73.1) | 4 (4.9) | 6 (6.9) | 2 (1.1) | 0 (0.0) | 0 (0.0) | 0.88 |
| McManus (1985) (ICM2pat) | 86 (85.9) | 4 (4.1) | 8 (8.1) | 1 (0.9) | 0 (0.0) | 0 (0.0) | 0.01 |
| Leiber & Axelrod (1981) | 1729 (1737.5) | 173 (164.5) | 281 (273.4) | 63 (70.6) | 8 (7.2) | 3 (3.8) | 1.8 |
| ^b^ These data are taken from McManus (1985). | | | | | | | |
| * A significant difference between model and data at p = 0.05. | | | | | | | |

**Table S6. Scenario B: Results of goodness-of-fit test with adjustment for estimates without adjustment with three parameters** $\hat{\boldsymbol{\rho}}$**= 0.267,** $\hat{\boldsymbol{\alpha}}$**= 0.148,** $\hat{\boldsymbol{\beta}}$ **= 0.012).** Model predictions are in parentheses, and G statistics for each study are in the right column. Model predictions fit the data in 16 of 17 individual studies and for all studies combined (G=43.6, df= 31, p<0.05).

**Table S7. Scenario B: Results of goodness-of-fit test with adjustment for estimates without adjustment with two parameters** $\hat{\boldsymbol{\rho}}$ **= 0.277,** $\hat{\boldsymbol{\alpha}}$ **= 0.138.** Model predictions are in parentheses, and G statistics for each study are in the right column. Model predictions fit the data in 16 of 17 individual studies and for all studies combined (G=44.4, df=32, p<0.05).

| Parental mating | Right x Right | | Right x Left | | Left x Left | |  |
| --- | --- | --- | --- | --- | --- | --- | --- |
| Offspring | Right | Left | Right | Left | Right | Left | G_df=1.88_ |
| Ramaley (1913) | 841 (826.7) | 115 (129.3) | 113 (123.3) | 54 (43.7) | 1 (4.9) | 7 (3.1) | 13.19* |
| Chamberlain (1928) | 6917 (6906.8) | 308 (318.2) | 411 (419.8) | 53 (44.2) | 18 (21.3) | 7 (3.7) | 5.1 |
| Rife (1940) | 1842 (1838.3) | 151 (154.7) | 140 (143.8) | 34 (30.2) | 5 (8.0) | 6 (3.0) | 4.37 |
| Merrell (1957) | 140 (137.8) | 34 (36.2) | 33 (37.2) | 20 (15.8) | 8 (6.1) | 2 (3.9) | 3.33 |
| Annett (1973) | 6206 (6214.0) | 669 (661.0) | 471 (469.9) | 125 (126.1) | 5 (4.0) | 1 (2.0) | 0.91 |
| Ferronato et al. (1947) | 154 (152.6) | 11 (12.4) | 31 (32.5) | 9 (7.5) | 0 (0.0) | 0 (0.0) | 0.54 |
| Mascie-Taylor (unpub)^b^ | 232 (232.7) | 17 (16.3) | 41 (40.3) | 7 (7.7) | 3 (3.0) | 1 (1.0) | 0.11 |
| Chaurasia & Goswani (unpub)^b^ | 1060 (1067.7) | 144 (136.3) | 122 (126.8) | 46 (41.2) | 3 (4.4) | 4 (2.6) | 2.27 |
| Annett (1978) | 1656 (1652.4) | 130 (133.6) | 170 (174.7) | 40 (35.3) | 4 (3.0) | 0 (1.0) | 3.26 |
| Carter-Saltzmann (1980) | 303 (301.8) | 37 (38.2) | 45 (45.5) | 15 (14.5) | 0 (0.0) | 0 (0.0) | 0.06 |
| Coren & Porac (1980) | 315 (320.5) | 68 (62.5) | 57 (52.1) | 16 (20.9) | 0 (0.0) | 0 (0.0) | 2.25 |
| McGee & Cozad (1980) | 848 (828.1) | 211 (230.9) | 325 (334.9) | 150 (140.1) | 30 (32.7) | 22 (19.3) | 3.8 |
| McManus (1985) (ICM1) | 58 (58.0) | 9 (9.0) | 14 (14.0) | 5 (5.0) | 0 (0.0) | 0 (0.0) | 0 |
| McManus (1985) (ICM2prop) | 134 (131.7) | 15 (17.3) | 17 (19.6) | 9 (6.4) | 1 (0.6) | 0 (0.4) | 2.61 |
| McManus (1985) (ICM2mat) | 74 (73.1) | 4 (4.9) | 6 (6.9) | 2 (1.1) | 0 (0.0) | 0 (0.0) | 0.88 |
| McManus (1985) (ICM2pat) | 86 (85.9) | 4 (4.1) | 8 (8.1) | 1 (0.9) | 0 (0.0) | 0 (0.0) | 0.01 |
| Leiber & Axelrod (1981) | 1729 (1737.5) | 173 (164.5) | 281 (273.1) | 63 (70.9) | 8 (7.4) | 3 (3.6) | 1.75 |
| ^b^ These data are taken from McManus (1985). | | | | | | | |
| * A significant difference between model and data at p = 0.05. | | | | | | | |

**Table S8. Scenario C: Results of goodness-of-fit test with adjustment for estimates with adjustment with three parameters** $\hat{\boldsymbol{\rho}}$**= 0.207,** $\hat{\boldsymbol{\alpha}}$**= 0.203,** $\hat{\boldsymbol{\beta}}$**= 0.042.** Model predictions are in parentheses, and G statistics for each study are in the right column. Model predictions fit the data in 16 of 17 individual studies and for all studies combined (G=36.3, df= 31, p<0.05).

| Parental mating | Right x Right | | Right x Left | | Left x Left | |  |
| --- | --- | --- | --- | --- | --- | --- | --- |
| Offspring | Right | Left | Right | Left | Right | Left | G_df=1.82_ |
| Ramaley (1913) | 841 (831.1) | 115 (124.9) | 113 (119.7) | 54 (47.3) | 1 (3.9) | 7 (4.1) | 7.1* |
| Chamberlain (1928) | 6917 (6908.2) | 308 (316.8) | 411 (418.7) | 53 (45.3) | 18 (20.6) | 7 (4.4) | 3.31 |
| Rife (1940) | 1842 (1839.3) | 151 (153.7) | 140 (143.2) | 34 (30.8) | 5 (7.5) | 6 (3.5) | 2.77 |
| Merrell (1957) | 140 (139.8) | 34 (34.2) | 33 (36.0) | 20 (17.0) | 8 (5.0) | 2 (5.0) | 4.63 |
| Annett (1973) | 6206 (6217.8) | 669 (657.2) | 471 (467.3) | 125 (128.7) | 5 (3.7) | 1 (2.3) | 1.8 |
| Ferronato et al. (1947) | 154 (152.7) | 11 (12.3) | 31 (32.5) | 9 (7.5) | 0 (0.0) | 0 (0.0) | 0.48 |
| Mascie-Taylor (unpub)^b^ | 232 (232.9) | 17 (16.1) | 41 (40.2) | 7 (7.8) | 3 (2.8) | 1 (1.2) | 0.2 |
| Chaurasia & Goswani (unpub)^b^ | 1060 (1075.0) | 144 (129.0) | 122 (123.6) | 46 (44.4) | 3 (3.5) | 4 (3.5) | 2.11 |
| Annett (1978) | 1656 (1653.4) | 130 (132.6) | 170 (174.1) | 40 (35.9) | 4 (2.8) | 0 (1.2) | 3.59 |
| Carter-Saltzmann (1980) | 303 (303.1) | 37 (36.9) | 45 (44.4) | 15 (15.6) | 0 (0.0) | 0 (0.0) | 0.04 |
| Coren & Porac (1980) | 315 (322.3) | 68 (60.7) | 57 (50.6) | 16 (22.4) | 0 (0.0) | 0 (0.0) | 3.77 |
| McGee & Cozad (1980) | 848 (840.4) | 211 (218.6) | 325 (327.0) | 150 (148.0) | 30 (28.2) | 22 (23.8) | 0.63 |
| McManus (1985) (ICM1) | 58 (58.4) | 9 (8.6) | 14 (13.6) | 5 (5.4) | 0 (0.0) | 0 (0.0) | 0.06 |
| McManus (1985) (ICM2prop) | 134 (132.4) | 15 (16.6) | 17 (19.1) | 9 (6.9) | 1 (0.5) | 0 (0.5) | 2.33 |
| McManus (1985) (ICM2mat) | 74 (73.1) | 4 (4.9) | 6 (6.9) | 2 (1.1) | 0 (0.0) | 0 (0.0) | 0.83 |
| McManus (1985) (ICM2pat) | 86 (85.9) | 4 (4.1) | 8 (8.1) | 1 (0.9) | 0 (0.0) | 0 (0.0) | 0.01 |
| Leiber & Axelrod (1981) | 1729 (1739.1) | 173 (162.9) | 281 (272.4) | 63 (71.6) | 8 (6.7) | 3 (4.3) | 2.68 |
| ^b^ These data are taken from McManus (1985). | | | | | | | |
| * A significant difference between model and data at p = 0.05. | | | | | | | |

**Table S9. Scenario C: Results of goodness-of-fit test with adjustment for estimates with adjustment with two parameters** $\hat{\boldsymbol{\rho}}$ **= 0.239,** $\hat{\boldsymbol{\alpha}}$ **= 0.172.** Model predictions are in parentheses, and G statistics for each study are in the right column. Model predictions fit the data in 16 of 17 individual studies and for all studies combined (G=37.5, df=32, p<0.05).

| Parental mating | Right x Right | | Right x Left | | Left x Left | |  |
| --- | --- | --- | --- | --- | --- | --- | --- |
| Offspring | Right | Left | Right | Left | Right | Left | G_df=1.88_ |
| Ramaley (1913) | 841 (831.5) | 115 (124.5) | 113 (118.9) | 54 (48.1) | 1 (4.4) | 7 (3.6) | 8.29* |
| Chamberlain (1928) | 6917 (6908.2) | 308 (316.8) | 411 (418.5) | 53 (45.5) | 18 (21.2) | 7 (3.8) | 4.22 |
| Rife (1940) | 1842 (1839.4) | 151 (153.6) | 140 (142.9) | 34 (31.1) | 5 (7.9) | 6 (3.1) | 3.79 |
| Merrell (1957) | 140 (140.1) | 34 (33.9) | 33 (35.6) | 20 (17.4) | 8 (5.4) | 2 (4.6) | 3.55 |
| Annett (1973) | 6206 (6217.9) | 669 (657.1) | 471 (466.4) | 125 (129.6) | 5 (4.0) | 1 (2.0) | 1.35 |
| Ferronato et al. (1947) | 154 (152.8) | 11 (12.2) | 31 (32.3) | 9 (7.7) | 0 (0.0) | 0 (0.0) | 0.4 |
| Mascie-Taylor (unpub)^b^ | 232 (233.0) | 17 (16.0) | 41 (40.1) | 7 (7.9) | 3 (2.9) | 1 (1.1) | 0.19 |
| Chaurasia & Goswani (unpub)^b^ | 1060 (1075.9) | 144 (128.1) | 122 (122.4) | 46 (45.6) | 3 (3.9) | 4 (3.1) | 2.65 |
| Annett (1978) | 1656 (1653.4) | 130 (132.6) | 170 (173.7) | 40 (36.3) | 4 (2.9) | 0 (1.1) | 3.04 |
| Carter-Saltzmann (1980) | 303 (303.2) | 37 (36.8) | 45 (44.1) | 15 (15.9) | 0 (0.0) | 0 (0.0) | 0.07 |
| Coren & Porac (1980) | 315 (322.4) | 68 (60.6) | 57 (50.3) | 16 (22.7) | 0 (0.0) | 0 (0.0) | 4.14 |
| McGee & Cozad (1980) | 848 (841.9) | 211 (217.1) | 325 (324.0) | 150 (151.0) | 30 (29.6) | 22 (22.4) | 0.24 |
| McManus (1985) (ICM1) | 58 (58.5) | 9 (8.5) | 14 (13.5) | 5 (5.5) | 0 (0.0) | 0 (0.0) | 0.09 |
| McManus (1985) (ICM2prop) | 134 (132.5) | 15 (16.5) | 17 (18.9) | 9 (7.1) | 1 (0.6) | 0 (0.4) | 1.98 |
| McManus (1985) (ICM2mat) | 74 (73.1) | 4 (4.9) | 6 (6.9) | 2 (1.1) | 0 (0.0) | 0 (0.0) | 0.81 |
| McManus (1985) (ICM2pat) | 86 (85.9) | 4 (4.1) | 8 (8.1) | 1 (0.9) | 0 (0.0) | 0 (0.0) | 0.01 |
| Leiber & Axelrod (1981) | 1729 (1739.5) | 173 (162.5) | 281 (271.2) | 63 (72.8) | 8 (7.3) | 3 (3.7) | 2.69 |
| ^b^ These data are taken from McManus (1985). | | | | | | | |
| * A significant difference between model and data at p = 0.05. | | | | | | | |

**Table S10. Twin data, model predictions, and goodness-of-fit results.** In the R-R, R-L, and L-L columns (for a pair of right, mixed, and left-handed twins), the value is the data, and the value in parentheses is the model prediction. Parameters estimated without adjustment, $\hat{\boldsymbol{\rho}}\mathbf{=0.277,}\hat{\boldsymbol{\alpha}}\mathbf{=0.138}$ (β fixed at zero). Model predictions fit the data in 27 out of the 28 individual studies and for all studies combined (G=35.68, df=28, p=0.151).

| Study | MZ twins | | | | | DZ twins | | | | |
| --- | --- | --- | --- | --- | --- | --- | --- | --- | --- | --- |
|  | $m_{o}$ | $R\times R$ | $R\times L$ | $L\times L$ | $G$  df=1 | $m_{o}$ | $R\times R$ | $R\times L$ | $L\times L$ | $G$  df=1 |
| Wilson & Jones (1932) | 0.1071 | 97 (97.0) | 24 (23.9) | 2 (2.0) | 0.0 | 0.1138 | 97 (97.0) | 24 (23.9) | 2 (2.0) | 0.0 |
| Stocks (1933) | 0.0952 | 76 (75.4) | 16 (17.3) | 2 (1.4) | 0.57 | 0.1064 | 76 (75.4) | 16 (17.3) | 2 (1.4) | 0.35 |
| Newman et al. (1937) | 0.19 | 39 (39.8) | 11 (9.4) | 0 (0.8) | 0.79 | 0.11 | 39 (39.8) | 11 (9.4) | 0 (0.8) | 1.82 |
| Bouterwek (1938) | 0.1885 | 23 (24.2) | 12 (9.7) | 0 (1.2) | 0.19 | 0.1714 | 23 (24.2) | 12 (9.7) | 0 (1.2) | 2.86 |
| Rife (1940) | 0.1188 | 104 (105.0) | 39 (37.0) | 3 (4.0) | 1.22 | 0.1541 | 104 (105.0) | 39 (37.0) | 3 (4.0) | 0.39 |
| Thyss (1946) | 0.1845 | 60 (60.6) | 24 (22.8) | 2 (2.6) | 3.62 | 0.1628 | 60 (60.6) | 24 (22.8) | 2 (2.6) | 0.21 |
| Rife (1950) | 0.1283 | 164 (165.7) | 45 (41.7) | 2 (3.7) | 0.19 | 0.1161 | 164 (165.7) | 45 (41.7) | 2 (3.7) | 1.18 |
| Dechaume (1957) | 0.2424 | 21 (21.4) | 11 (10.2) | 1 (1.4) | 0.0 | 0.197 | 21 (21.4) | 11 (10.2) | 1 (1.4) | 0.18 |
| Zazzo (1960) | 0.1332 | 264 (267.1) | 69 (62.8) | 2 (5.1) | 2.66 | 0.109 | 264 (267.1) | 69 (62.8) | 2 (5.1) | 3.11 |
| Carter-Saltzmann et al. (1976) | 0.1711 | 115 (115.1) | 54 (53.7) | 7 (7.1) | 1.9 | 0.1932 | 115 (115.1) | 54 (53.7) | 7 (7.1) | 0.0 |
| Loehlin & Nichols (1976) | 0.1411 | 261 (264.3) | 70 (63.4) | 2 (5.3) | 0.16 | 0.1111 | 261 (264.3) | 70 (63.4) | 2 (5.3) | 3.39 |
| Springer & Searleman (1978) | 0.1667 | 35 (33.4) | 9 (12.3) | 3 (1.4) | 0.26 | 0.1596 | 35 (33.4) | 9 (12.3) | 3 (1.4) | 2.49 |
| NCDS (unpublished) | 0.1512 | 66 (64.2) | 18 (21.5) | 4 (2.2) | 0.85 | 0.1477 | 66 (64.2) | 18 (21.5) | 4 (2.2) | 1.77 |
| Neale (1988) | 0.122 | 626 (621.9) | 183 (191.2) | 23 (18.9) | 4.3* | 0.1376 | 626 (621.9) | 183 (191.2) | 23 (18.9) | 1.22 |

**Table S11.** Probabilities of right-handed female offspring (top) and right-handed male offspring (bottom) given offspring genotype (columns) and parental phenotypes (columns). The full recursion is given in supplementary text S8.

| Parents of female offspring | DD | DC | CC |
| --- | --- | --- | --- |
| R x R | (1*/*2) + *ρ* + *α_F_* | (1*/*2) + *h*_1_*ρ* + *α_F_* | (1*/*2) + *α_F_* |
| R x L | (1*/*2) + *ρ* + *β_F_* | (1*/*2) + *h*_1_*ρ* + *β_F_* | (1*/*2) + *β_F_* |
| L x R | (1*/*2) + *ρ* + *γ_F_* | (1*/*2) + *h*_1_*ρ* + *γ_F_* | (1*/*2) + *γ_F_* |
| L x L | (1*/*2) + *ρ* − *α_F_* | (1*/*2) + *h*_1_*ρ* − *α_F_* | (1*/*2) − *α_F_* |

| Parents of male offspring | DD | DC | CC |
| --- | --- | --- | --- |
| R x R | (1*/*2) + *ρ* + *α_M_* | (1*/*2) + *h*_1_*ρ* + *α_M_* | (1*/*2) + *α_M_* |
| R x L | (1*/*2) + *ρ* + *β_M_* | (1*/*2) + *h*_1_*ρ* + *β_M_* | (1*/*2) + *β_M_* |
| L x R | (1*/*2) + *ρ* + *γ_M_* | (1*/*2) + *h*_1_*ρ* + *γ_M_* | (1*/*2) + *γ_M_* |
| L x L | (1*/*2) + *ρ* − *α_M_* | (1*/*2) + *h*_1_*ρ* − *α_M_* | (1*/*2) − *α_M_* |

**Table S12.** Maximum-likelihood estimates for parameters of the extended models for the McKeever dataset. * for fixed values (not estimated).

|  | I | II | III | IV | V |
| --- | --- | --- | --- | --- | --- |
| *α_F_* | 0.0656 | 0.0230 | 0.0230 | 0.0335 | 0.0335 |
| *α_M_* | *α_F_** | *α_F_** | *α_F_** | 0.0163 | 0.0163 |
| *β_F_* | 0* | -0.0482 | -0.0159 | -0.035 | 0.0027 |
| *β_M_* | 0* | *β_F_** | *β_F_** | -0.058 | -0.0309 |
| *γ_F_* | 0* | *β_F_** | -0.0918 | *β_F_** | -0.085 |
| *γ_M_* | 0* | *β_F_** | *γ_F_** | *β_M_** | -0.095 |
| *ρ* | 0.324 | 0.3671 | 0.3671 | 0.3655 | 0.3655 |
| S_T_(θ\|D) | -7726.3 | -7723.6 | -7710.8 | -7715.4 | -7701.9 |
|  |  |  |  |  |  |

**Table S13.** Maximum-likelihood estimates for parameters of the extended models for Generation 1 from Nurhayu et al. * for fixed values (not estimated).

|  | I | II | III | IV | V |
| --- | --- | --- | --- | --- | --- |
| *α_F_* | 0.1198 | 0.1574 | 0.1025 | 0.1422 | .1143 |
| *α_M_* | *α_F_* | *α_F_* | *α_F_* | 0.1173 | .0894 |
| *β_F_* | - | -0.1259 | -0.0121 | -0.0009 | 0.0195 |
| *β_M_* | - | *β_F_* | *β_F_* | -0.1896 | -0.1990 |
| *γ_F_* | - | *β_F_* | -0.1335 | *β_F_* | -0.1488 |
| *γ_M_* | - | *β_F_* | *γ_F_* | *β_M_* | -0.2815 |
| *ρ* | 0.3165 | 0.3283 | 0.2360 | 0.3478 | 0.3757 |
| S_T_(θ\|D) | -1062.99 | -1062.90 | -1061.28 | -1052.02 | -1049.62 |

**Table S14.** Maximum-likelihood estimates for parameters of the extended models for Generation 2 from Nurhayu et al. * for fixed values (not estimated).

|  | I | II | III | IV | V |
| --- | --- | --- | --- | --- | --- |
| *α_F_* | 0.1336 | 0.1891 | 0.1426 | 0.0500 | .0730 |
| *α_M_* | *α_F_* | *α_F_* | *α_F_* | 0.0092 | .0320 |
| *β_F_* | - | -0.2857 | -0.2031 | -0.0924 | -0.0602 |
| *β_M_* | - | *β_F_* | *β_F_* | -0.2002 | -0.1745 |
| *γ_F_* | - | *β_F_* | -0.2488 | *β_F_* | -0.0850 |
| *γ_M_* | - | *β_F_* | *γ_F_* | *β_M_* | -0.1759 |
| *ρ* | 0.3163 | 0.2801 | 0.3039 | 0.4400 | 0.4166 |
| S_T_(θ\|D) | -830.24 | -829.41 | -829.30 | -817.66 | -817.61 |

**Table S15.** Representation of the Indonesian datasets (Nurhayu et al., 2020) as triplets.

|  | p (L_m_)  progeny | p (L_m_)  parental | R×R | | R×L | | L×L | |
| --- | --- | --- | --- | --- | --- | --- | --- | --- |
|  |  |  | R | L | R | L | R | L |
| Generation 1 | 0.0823 | 0.0851 | 2707 | 184 | 438 | 94 | 19 | 6 |
| Generation 2 | 0.0875 | 0.0558 | 1439 | 106 | 178 | 48 | 10 | 2 |

# Supplementary Figures


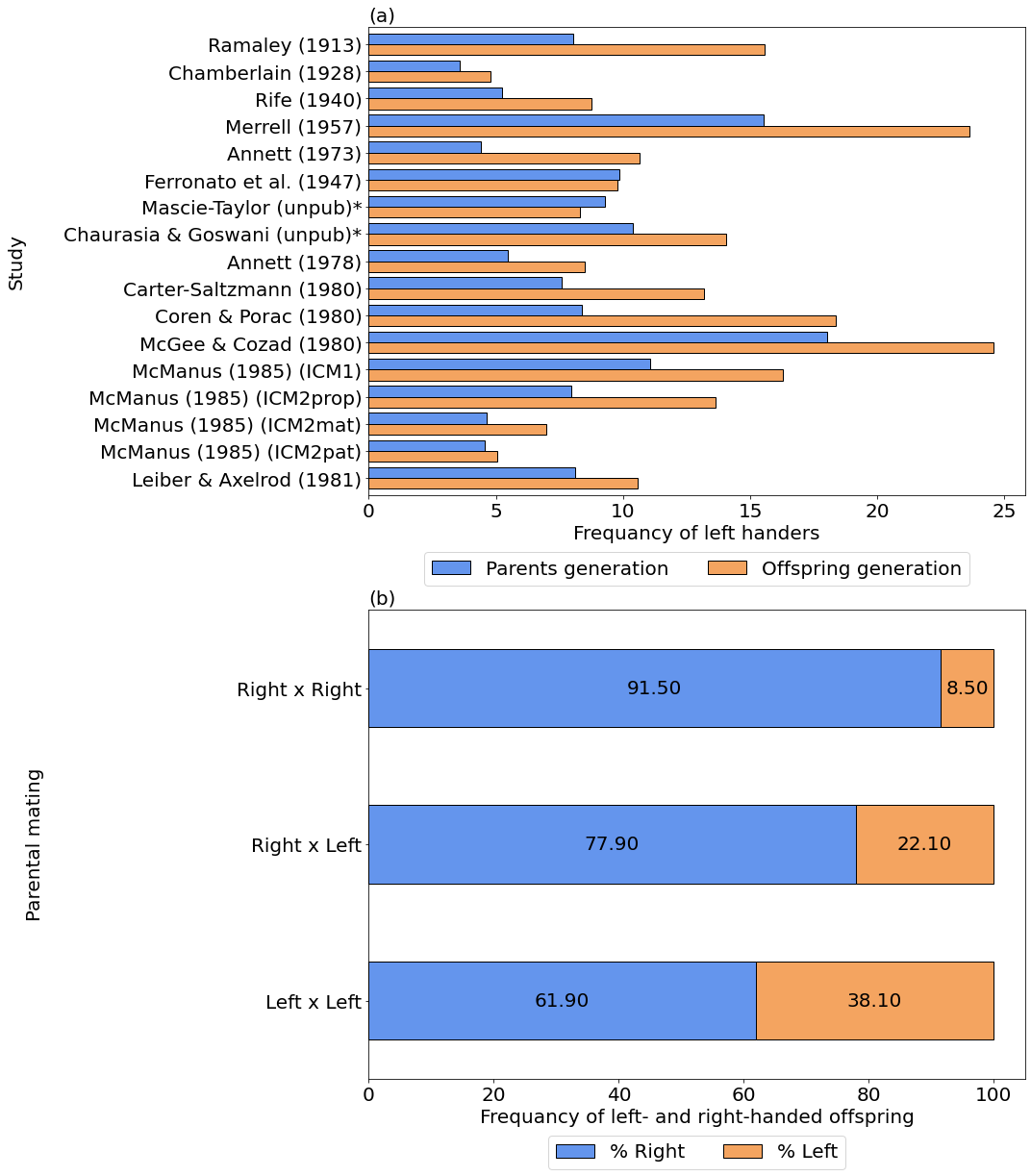


**Figure S1. Frequency of left-handers reported by Laland et al. (1995).** (a) The frequency of left-handers in the 17 studies by generation. (b) Frequency of left- and right-handers by parental mating.

*These data are taken from McManus (1985).

**
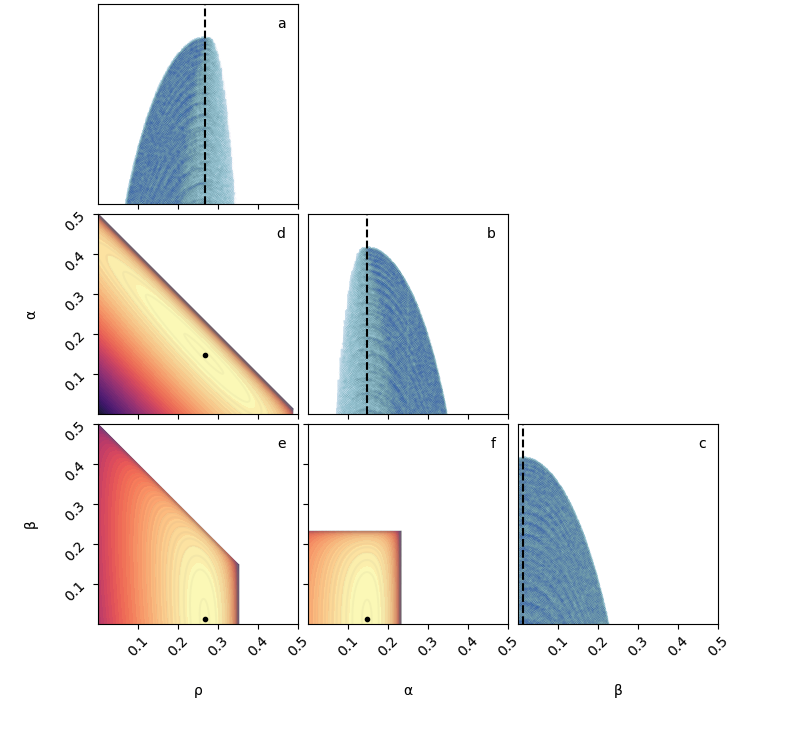
**

**Figure S2. Scenarios A and B: Likelihood estimation of** $\boldsymbol{\rho}$**,** $\boldsymbol{\alpha}$**, and** $\boldsymbol{\beta}$ **without adjustment.** Maximum likelihood estimates are $\hat{\rho}=0.267, \hat{\alpha}=0.148$ and $\hat{\beta}=0.012$ with a log-likelihood of -8826.643. Diagonal panels (a, b, c): Markers show log-likelihood values across a range of 1,000 parameter values (1,000^3^ combinations of $\rho, \alpha$ and $\beta$). Vertical dashed lines indicate the maximum likelihood estimates for (a) the effect of genetic transmission parameter $\rho$, (b) the effect of same-handed parents parameter $\alpha$, and (c) the effect of mixed-handed parents parameter $\beta$. Corner panels (d, e, f): Contour plot shows the joint log-likelihood surfaces for pairs of parameters. Markers indicate point estimates for ρ, α, and β. Point estimates were estimated using the Nelder-Mead algorithm, while the contour plots show results of computing the log-likelihood over a grid with 1,000 values for each parameter. Point estimates obtained from both methods were effectively identical.

**
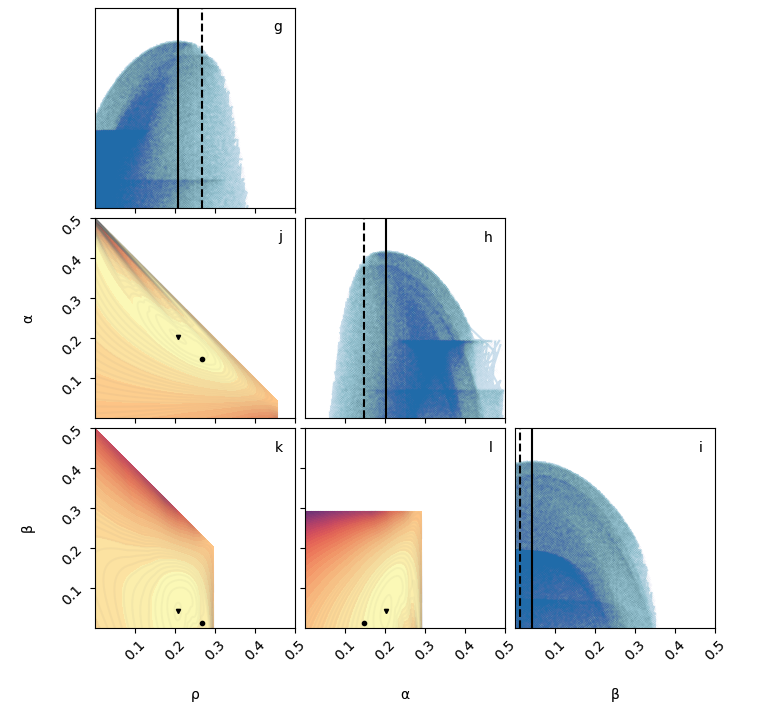
**

**Figure S3. Scenario C: Likelihood estimation of** $\boldsymbol{\rho}$**,** $\boldsymbol{\alpha}$**, and** $\boldsymbol{\beta}$ **with adjustment.** Maximum likelihood estimates are $\hat{\rho}=0.207, \hat{\alpha}=0.203,$and $\hat{\beta}=0.042$ with log-likelihood of -8566.939. Diagonal panels (g, h, i): Markers show log-likelihood values across a range of 1,000 parameter values (1,000^3^ combinations of $\rho, \alpha$ and $\beta$). Dashed and solid lines indicate the maximum likelihood estimates without and with adjustment, respectively, for (g) the effect of genetic transmission parameter $\rho$, (h) the impact of same-handed parents parameter $\alpha$, and (j) the effect of mixed-handed parents parameter $\beta$. Corner panels: (d, e, f): Contour plot shows the joint log-likelihood surfaces for pairs of parameters. Point estimates with and without adjustment are indicated by triangles and circles, respectively. Point estimates were estimated using the Nelder-Mead algorithm, while the contour plots show results of computing the log-likelihood over a grid with 1,000 values for each parameter. Point estimates obtained from both methods were effectively identical.

*
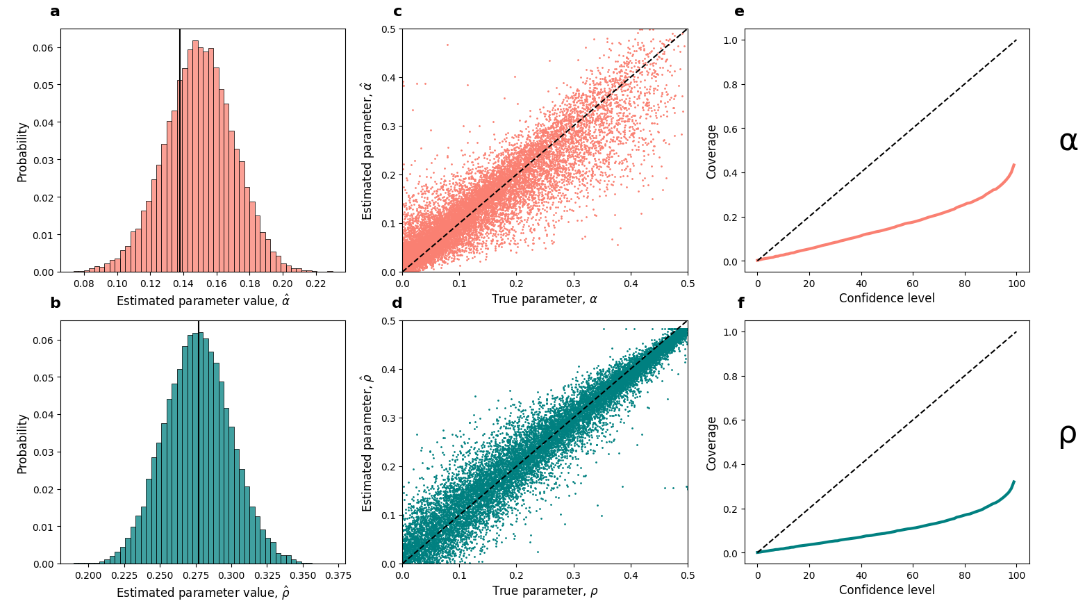
*

**Figure S4. Performance of the estimation method without adjustment and with linear regression correction on simulated synthetic data.** This figure reproduces Figure 3 from the main text with an additional step in which the estimates are corrected using a linear regression model. **(a, b)** The distribution of $\hat{\alpha}$ and $\hat{\rho}$ estimated from data simulated with the values estimated by Laland et al. (solid lines; ρ=0.277 in panel a and α=0.138 in panel b). **(c, d)** Scatter plot of parameter estimates (y-axis) vs. the true parameter (x-axis). **(e, f)** Coverage for various confidence levels: the rate at which the true parameter value falls within the estimated confidence interval at a given confidence level (higher is confidence level gives a wider confidence interval). Confidence interval computed by non-parametric bootstrap with 200 resamples.

*
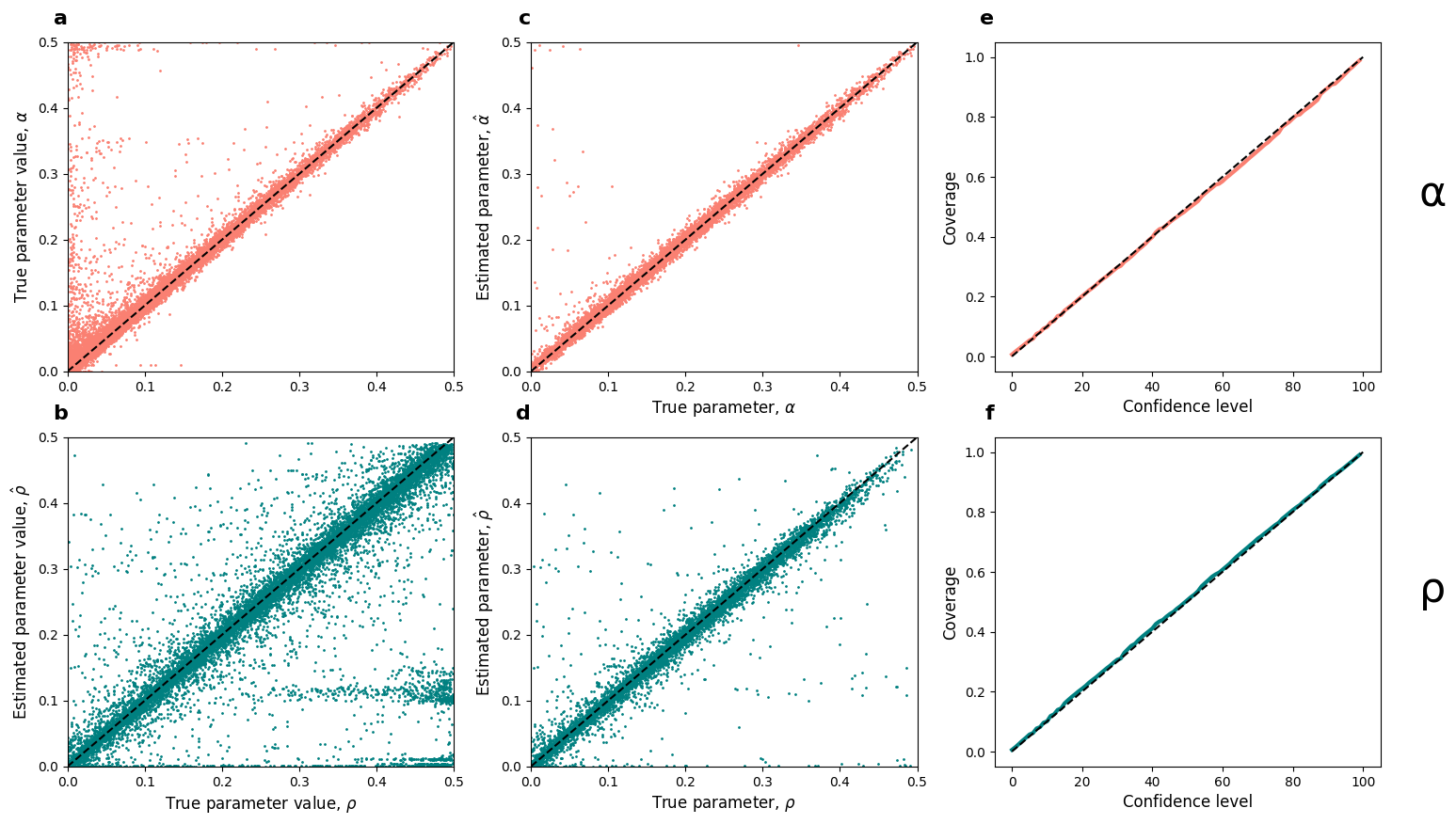
***Figure S5. Performance of the estimation method with adjustment on simulated synthetic data.** Compare to panels c-f in Figure 3. **(a, b)** Scatter plot of parameter estimates (y-axis) vs. the true parameter (x-axis). **(c, d)** Similar to panels a and b, respectively, after filtering synthetic data in which *m_o_* ≤ 0.01 or *m_p_*≤0.01 or *|p(R|R×R) - |p(R|L×L)|*<0.07. **(e, f)** Coverage for various confidence levels: the rate at which the true parameter value falls within the estimated confidence interval at a given confidence level (higher confidence level gives a wider confidence interval). Confidence interval computed by non-parametric bootstrap with 200 resamples.


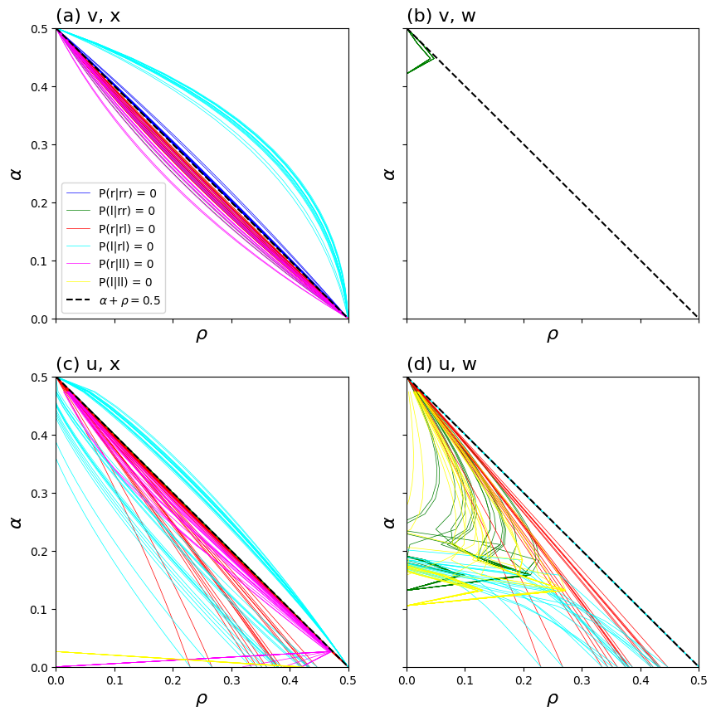


**Figure S6. Ruggedness of the adjusted log-likelihood function.** The figure shows the adjusted log-likelihood $S_{M}(\rho,\alpha|D)$ (eq. 5) of the two-parameter model as a function of the model parameters. Color curves show paths in the parameter space where one of the conditional probabilities (*P(offspring phenotype | parent mating type)=0*, see legend) is zero, so one of the terms in $S_{M}$ (eq. 5) is log of zero and $S_{M}$ is negative infinity. Variation between the lines is due to different *m_p_* and *m_o_* values for each dataset.
